# The HD-ZIP IV transcription factor GL2-LIKE regulates male flowering time and fertility in cucumber

**DOI:** 10.1093/jxb/eraa251

**Published:** 2020-06-03

**Authors:** Yanling Cai, Ezra S Bartholomew, Mingming Dong, Xuling Zhai, Shuai Yin, Yaqi Zhang, Zhongxuan Feng, Licai Wu, Wan Liu, Nan Shan, Xiao Zhang, Huazhong Ren, Xingwang Liu

**Affiliations:** 1 Beijing Key Laboratory of Growth and Developmental Regulation for Protected Vegetable Crops, College of Horticulture, China Agricultural University, Beijing, P. R. China; 2 Engineering Research Center of Breeding and Propagation of Horticultural Crops, Ministry of Education, College of Horticulture, China Agricultural University, Beijing, P. R. China; 3 Trinity College Dublin, Ireland

**Keywords:** Cucumber, HD-ZIP IV–CsJAZ1 complex, HD-ZIP IV transcription factor, jasmonate, jasmonate repressor, male flowering

## Abstract

Cucumber is dioecious by nature, having both male and female flowers, and is a model system for unisexual flower development. Knowledge related to male flowering is limited, but it is reported to be regulated by transcription factors and hormone signals. Here, we report functional characterization of the cucumber (*Cucumis sativus*) *GL2-LIKE* gene, which encodes a homeodomain leucine zipper (HD-ZIP) IV transcription factor that plays an important role in regulating male flower development. Spatial–temporal expression analyses revealed high-level expression of *CsGL2-LIKE* in the male flower buds and anthers. CsGL2-LIKE is closely related to AtGL2, which is known to play a key role in trichome development. However, ectopic expression of *CsGL2-LIKE* in Arabidopsis *gl2-8* mutant was unable to rescue the *gl2-8* phenotype. Interestingly, the silencing of *CsGL2-LIKE* delayed male flowering by inhibiting the expression of the florigen gene *FT* and reduced pollen vigor and seed viability. Protein–protein interaction assays showed that CsGL2-LIKE interacts with the jasmonate ZIM domain protein CsJAZ1 to form a HD-ZIP IV–CsJAZ1 complex. Collectively, our study indicates that CsGL2-LIKE regulates male flowering in cucumber, and reveals a novel function of a HD-ZIP IV transcription factor in regulating male flower development of cucumber.

## Teaser

CsGL2-LIKE, a HD-ZIP IV transcription factor, regulates male flowering time and fertility in cucumber and interacts with the jasmonate repressor CsJAZ1 to form a HD-ZIP IV–CsJAZ1 complex.

## Introduction

The transition from vegetative to reproductive growth, generally called flowering, is a critical developmental change that influences plant productivity. Flowering is a finely tuned process that is regulated by several endogenous and exogenous factors, such as plant age, photoperiod, vernalization, and phytohormones (Liu *et al.*, 2009; Wellmer and Riechmann, 2010). In Arabidopsis, inputs from internal and environmental flowering pathways are integrated by a core set of floral pathway integrator genes (Simpson & Dean, 2002), such as *FLOWERING LOCUS T* (*FT*: At1g65480), which encodes a small globular protein named florigen. FT accumulates in the shoot apical meristem (SAM) and promotes the identity switching of the SAM from vegetative to reproductive (Srikanth and Schmid, 2011; Song *et al.*, 2013). Under optimal flowering conditions, *FT* expression is positively regulated by *CONSTANS* (*CO*: At5g15840), which encodes a putative zinc finger transcription factor (TF) (Putterill *et al.*, 1995; Fornara *et al.*, 2009; Zhai *et al.*, 2015). FT also interacts with *FLOWERING LOCUS D* (*FD*: At4g35900) to promotes the expression of several downstream genes, including the floral meristem identity genes *APETALA1* (*AP1*: At1g69120), *FRUITFULL* (*FUL*: At5g60910), and *LEAFY* (*LFY*: At5g61850) (Abe *et al.*, 2005; Wigge *et al.*, 2005; Corbesier *et al.*, 2007; Amasino, 2010). Under unfavorable conditions for flowering, the expression of *FT* is negatively regulated by several transcriptional repressors, such as FLOWERING LOCUS C (Helliwell *et al.*, 2006; Searle *et al.*, 2006), SHORT VEGETATIVE PHASE (Lee *et al*., 2007), TEMPRANILLO1 (Castillejo and Pelaz, 2008), and SCHLAFMŰTZE (SMZ) (Mathieu *et al*., 2009). Together, these studies emphasize the central role of FT in mediating the crosstalk between environmental stress signaling and the internal genetic flowering network.

Jasmonic acid and its derivatives, commonly referred to as jasmonates (JAs), are good examples of signaling molecules that modulate a wide range of developmental processes, including flowering time, in response to ever-changing environmental conditions. Recent progress in dissecting the JA signaling pathway has revealed that upon environmental or endogenous flowering stimuli, plants generate jasmonic acid–isoleucine (JA-Ile), which specifically promotes the assembly of a co-receptor composed of CORONATINE INSENSITIVE1 (COI1), an F-box protein component of the E3 ubiquitin ligase SCF^COI1^, and transcriptional repressors of the JASMONATE ZIM-DOMAIN (JAZ) protein family (Chini et al., 2007, 2016). In the presence of JA-Ile, COI1 binds to JAZs, leading to the ubiquitination and subsequent degradation of the JAZ proteins by the 26S proteasome (Thines *et al.*, 2007; Chini et al., 2007, 2016; Sheard *et al.*, 2010; Wasternack and Hause, 2013). This induces the expression of several early JA-responsive genes, such as *JAZ*s and *MYC*s (Chini et al., 2007, 2016; Chung *et al.*, 2008). *JAZ*s modulate several floral development-related TFs, including the MYC2/3/4/5 proteins that regulate flower development and seed production (Fernández-Calvo *et al.*, 2011; Schweizer *et al.*, 2013; Qi *et al.*, 2015; Wang *et al.*, 2017), the AP2 family TFs TARGET OF EAT1 (TOE1) and TOE2 that repress the transcription of *FT* (Zhai *et al.*, 2015), and the R2R3-MYB TFs MYB21 and MYB24 that influence stamen fertility in Arabidopsis (Cheng *et al.*, 2011; Song *et al.*, 2011; Boter *et al.*, 2015). In the absence of JA-Ile, JAZs along with several associated co-repressors bind to and block TFs (e.g. MYC2/3/4) that target downstream genes involved in specific JA responses (Wasternack and Hause, 2013; Gimenez-Ibanez *et al.*, 2015; Chini *et al.*, 2016).

The homeodomain leucine zipper (HD-ZIP) family is a group of related developmental TFs that are known to mediate the action of phytohormones and are involved in responses to environmental stimuli (Ariel *et al.*, 2007). The HD-ZIP IV subfamily, also named HD-ZIP GL2 after the Arabidopsis GLABRA2 (GL2) protein, plays a central role in epidermal cell differentiation and reproductive organ development (Schrick *et al.*, 2004; Nakamura *et al.*, 2006; Ariel *et al.*, 2007). The Arabidopsis HD-ZIP IV family consists of 16 members; among them, two closely related and functionally redundant genes, *ARABIDOPSIS THALIANA MERISTEM LAYER1* (*ATML1*) and *PROTODERMAL FACTOR2* (*PDF2*), have been implicated in the regulation of embryo and epidermis development as well as the determination of floral identity (Kamata *et al.*, 2013; Ogawa *et al.*, 2015). Disruption of both *ATML1* and *PDF2* results in severe defects in shoot epidermal cell differentiation (Abe *et al.*, 2003). In the double-mutant of *pdf2-1* with homeodomain glabrous 2–3 (*hdg2-3*), flowers with sepaloid petals and carpelloid stamens were observed. Likewise, the *pdf2-1 hdg1-1*, *pdf2-1 hdg5-1*, and *pdf2-1 hdg12-2* mutants also exhibit abnormal petal and stamen formation (Kamata *et al.*, 2013). Functional analysis of some of the HD-ZIP IV genes has also been carried out in other plants such as maize, rice, and tomato. In maize, cuticle deposition and kernel development were reported to be regulated by the HD-ZIP IV transcription factor ZmOCL1 (HD-ZIP IV1), while ZmOCL4 (HD-ZIP IV4) was implicated in regulation of development of anther wall as well as trichomes (Vernoud *et al.*, 2009; Javelle *et al.*, 2010).

Cucumber (*Cucumis sativus* L., 2*n*=2*x*=14) is one of the most economically important vegetable crops in the world, and is the main vegetable grown in protected environments in China (Yang *et al.*, 2018). The cucumber flower is a well-established model system for unisexual flower development (Boualem *et al.*, 2015). Two *FT* homologs have been reported to influence flowering in cucumber (Sato *et al.*, 2009; Lu *et al.*, 2014), but the genetic and molecular mechanisms regulating flowering are still not clear. In a recent study, Yan *et al.* (2017) reported that JAZ protein interacts with a HD-ZIP IV protein to repress its transcriptional activity and that it regulates glandular trichome initiation. Here, we identified *CsGL2-LIKE*, a HD-ZIP IV TF that affects male fertility by reducing pollen vigor and producing unviable pollen tubes. The down-regulation of *CsGL2-LIKE* is associated with a delayed flowering of the male flower. We highlighted a molecular link between JAZs and the homeodomain regulator in controlling male flowering in cucumber.

## Materials and methods

### Plant materials and growth conditions

Cucumber (*C. sativus* L) inbred line 3461 (wild type, monoecious), a northern-type cucumber obtained from China Agricultural University (CAU; Liu *et al.*, 2016), was used for gene cloning, spatiotemporal expression analysis, and *Agrobacterium*-mediated transformation. Cucumber seedlings were grown at CAU Experimental Field Station, Beijing, under standard greenhouse conditions. The Arabidopsis mutant *gl2-8* was obtained from the Arabidopsis Biological Resource Center (Ohio State University, Columbus OH, USA; http://abrc.osu.edu/). The growth conditions for the *gl2-8* mutant and wild-type Columbia (Col) were 22 °C with a light cycle of 16 h light and 8 h dark. Cucumber seedling for phytohormone experiments and *Nicotiana benthamiana* seedlings were grown at 24±2 °C with a light cycle of 16 h light and 8 h dark.

### Quantitative real-time PCR analysis and cloning

Total RNA from various cucumber tissues was extracted using the Quick RNA plant isolation kit (Beijing Yueyang Biotechnology Ltd, Beijing, China), and cDNA was synthesized using a TIANScript II RT Kit (Tiangen Biotech, Beijing, China) following the manufacturers’ protocol. Quantitative real-time PCR (qRT-PCR) was conducted in 96-well plates with an ABI 7500 Real-Time PCR System (Applied Biosystems, USA) using the SYBR^®^ Premix Ex Taq (TaKaRa, Beijing, China). Gene expression was normalized relative to *Actin* (Csa6M484600) by using ΔΔ*C*_t_ method (Liu *et al.*, 2016). The cDNA of male flower buds was used for *CsGL2-LIKE* cloning. Gene specific primers are listed in Supplementary Table S1 and gene information is listed in Supplementary Table S2 at *JXB* online.

### Phylogenetic analysis

To study evolutionary relationships, the putative amino acid sequences of HD-ZIP IV protein sequences, from *C. sativus*, Arabidopsis and other species, obtained from the NCBI database (http://www.ncbi.nlm.nih.gov/BLAST/), were first aligned using CLUSTAL W (Thompson *et al.*, 1994) with default settings to construct an unrooted neighbor-joining (NJ) phylogenetic tree with Poisson correction using MEGA 5 (Saitou and Nei, 1987). Bootstrap analysis was employed using 15 000 replicates. The pair-wise gap deletion mode was used to ensure that the more divergent C-terminal domains could contribute to the topology of the NJ tree. The boxes were drawn using the BoxShade website (http://www.ch.embnet.org/software/BOX_form.html).

### Subcellular localization

To investigate the subcellular location of *CsGL2-LIKE* and *CsJAZ1*, the full-length coding regions of both *CsGL2-LIKE* and *CsJAZ1* without the stop codon were amplified using primers (see Supplementary Table S1) and inserted into the binary vector pCAMBIA1300 (CAMBIA) with a green fluorescent protein (GFP) tag between the *Sma*I and *Spe*I sites (Li *et al.*, 2017). These fusion proteins were transiently expressed in onion and tobacco epidermal cells as previously described (Varagona *et al.*, 1992; Schütze *et al.*, 2009). Images were taken with a Zeiss LSM 510 confocal laser-scanning microscope at an excitation wavelength of 488 nm.

### *In situ* RNA hybridization

*In situ* RNA hybridization was performed as previously described (Zhang *et al.*, 2013). Cucumber male and female flower buds, ovaries and shoot apexes of 16-day-old seedlings were harvested and fixed in 3.7% formalin–acetic acid–alcohol (FAA) solution and hybridized with digoxigenin-labeled probes. Specific regions of *CsGL2-LIKE* (1541–2346 bp) and *CsJAZ1* (1–444 bp) were selected for synthesis of probes with Sp6 and T7 tails. Gene-specific primers are listed in Supplementary Table S1.

### Generation of transgenic plants

#### Cucumber

A 260 bp fragment of the *CsGL2-LIKE* coding sequence was amplified and inserted in the pFGC1008 vector between the *Asc*I (5′ end) and *Swa*I (3′ end) restriction sites and the of *Spe*I (5′ end) and *Bam*HI (3′ end) sites to generate a *CsGL2-LIKE* RNAi vector (Chen *et al.*, 2016). After confirmation by sequencing, the recombinant plasmid was transformed into *Agrobacterium tumefaciens* strain C58 (Yang *et al.*, 2018). The recombinant plasmids were then transformed into cucumber inbred line 3461 via *Agrobacterium*-mediated transformation as previously described (Liu *et al.*, 2016).

#### Arabidopsis

The full-length *CsGL2-LIKE* or *AtGL2* coding region was amplified and inserted into pCAMBIA1300 (CAMBIA). The constructs were then introduced into *A. tumefaciens* strain GV3101 by electroporation, and the bacteria were subsequently transformed into Arabidopsis wild type (Col) as previously described (Clough & Bent, 1998). The transgenic plants were screened on Murashige and Skoog (MS) medium supplemented with 25 mg l^−1^ hygromycin (Sigma). Gene-specific primers are listed in Supplementary Table S1.

### RNA-seq and differentially expressed gene analysis

Total RNA from male flowers (three biological replicates) at −6 d post-anthesis (DPA) was extracted for RNA sequencing (RNA-seq) analyses (Sangon Biotech Co., Ltd, Shanghai). The expression of each gene was calculated and normalized to the fragments per kilobase of transcript per million mapped reads (FPKM). The false discovery rate (FDR) was used to determine the threshold of the *P*-value in multiple tests. In our study, an FDR <0.05 and a fold change >2 were used as significant cutoff values for expression differences.

### Yeast two-hybrid assays

Total RNA from male flower buds and ovaries was used to construct a yeast library (Oebiotech, Shanghai, China). The *CsGL2-LIKE* cDNA was inserted between the *Nde*I and *Eco*RI restriction sites of the pGBKT7 vector. Yeast library screening was performed using pGBKT7-CsGL2-LIKE as bait according to the manufacturer’s instructions (Clontech, Beijing). To confirm the results of the yeast library screening, we inserted *CsJAZ1* between the *Nde*I and *Eco*RI restriction sites of the pGADT7 vector. These two constructs were then co-transformed into yeast strain AH109. The co-transformation of pGBKT7-CsGL2-LIKE and a pGADT7 empty vector was used as a negative control. The transformants were cultivated on SD/− Leu/−Trp medium and tested on SD/−Ade/−Leu/−His/−Trp/X-α-Gal medium. Gene-specific primers are listed in Supplementary Table S1.

### Bimolecular fluorescence complementation analysis

The full lengths of *CsGL2-LIKE* and *CsJAZ1* were inserted into the *Asc*I and *Bam*HI restriction sites of pSPYNE and pSPYCE vectors to create CsGL2-LIKE-NE, CsGL2-LIKE-CE, CsJAZ1-NE, and CsJAZ1-CE (Walter *et al.*, 2004). The resulting constructs were then co-transformed into *A. tumefaciens* strain GV3101, which was subsequently transiently transformed into the leaves of 5-week-old *N. benthamiana* plants (Schütze *et al.*, 2009). GFP fluorescence was detected using a Zeiss LSM 510 confocal laser-scanning microscope with 488 nm laser light after the plants were cultivated for 48–72 h. CsGL2-LIKE-NE/pSPYCE, CsJAZ1-CE/pSPYNE and CsGL2-LIKE-NE/CsMYC2-CE co-transformations were used as negative controls. Gene-specific primers are listed in Supplementary Table S1.

### Co-immunoprecipitation assays

For co-immunoprecipitation assays, the full length of *CsGL2-LIKE-GFP* and CsJAZ1-3*HA was inserted between the *Eco*RI and *Bam*HI restriction sites of pCMBIA1300 (CAMBIA) to create 35S::CsGL2-LIKE-GFP and 35S::CsJAZ1-3*HA. These two vectors were subsequently co-transformed into *N. benthamiana* leaves as described before (Schütze et al., 2009). CsGL2-LIKE-GFP/pCMBIA1300-HA and CsJAZ1-HA/pCMBIA1300-GFP were used as negative controls. Leaf samples were collected after the plants were cultivated for 72 h, and total proteins were extracted by using lysis buffer (50 mM Tris-HCl (pH 7.4), 150 mM NaCl, 10% glycerol, 1 mM EDTA, and 0.5% Nonidet P-40). To inhibit protein degradation, a protease inhibitor cocktail consisting of 20 μM proteasome and inhibitor MG132 was used. The protein mixture was incubated with GFP antibody (Abclonal, China) and Protein G Sepharose (GE Healthcare, UK). Additionally, hemagglutinin (HA) antibody (Abclonal, China) was used in western blots for protein detection. Gene-specific primers are listed in Supplementary Table S1.

### Hormone treatment

We conducted hormone treatments using 50 μM methyl jasmonate (MeJA) with 1% ethanol used as a mock treatment. Cucumber line 3461 was grown on MS medium for 2 weeks, after which it was sprayed with 50 ml of hormone. Samples were collected at 0, 6, 12, 24, 48, and 72 h after hormone application (six plants constituted one replication). RNA was extracted from whole plants and qRT-PCR used to determine *CsGL2-LIKE* expression levels. The primers used are listed in Supplementary Table S1.

### JA and JA-Ile content measurement

The JA and JA-Ile content level of 0 DPA male flowers was measured using ultra-fast liquid chromatography–electrospray ionization tandem mass spectrometry according to the method of Liu *et al* (2012).

### Flowering time, *in vitro* pollen tube elongation tests and pollen vigor assays

To record the flowering time of male flowers, the first flower and floral growth were observed until anthesis. The observations were conducted in autumn 2016, spring 2017, and autumn 2017.

To test pollen tube elongation, pollen was obtained from 0 DPA male flowers and spread onto medium consisting of 10% (w/v) sucrose, 0.5% (w/v) boric acid, and 0.5% (w/v) Phytagel (Sigma) at room temperature. At least 20 dimensions of the images were obtained with an Olympus BX53 microscope taken after 45–60 min. To test pollen vigor, pollen was obtained from 0 DPA male flowers. The pollen was spread onto medium consisting of 1% (w/v) triphenyltetrazolium chloride solution (0.2 g of triphenyltetrazolium chloride and 12 g of sucrose dissolved in 20 ml of distilled water). The sample was kept at room temperature for 2 h and then examined with an Olympus BX53 microscope.

### Statistical analysis

Student’s *t*-test was applied by using the algorithm embedded in Microsoft Excel, and significance was determined by the same metric (*P*<0.05 or *P*<0.01).

## Results

### *CsGL2-LIKE* encodes a typical HD-ZIP IV transcription factor

In previous studies, Arabidopsis *GLABRA2* (*AtGL2*; AT1G79840) was isolated as a putative gene involved in epidermal cell determination (Di Cristina *et al.*, 1996). Mutations in *AtGL2* are known to affect several processes including mucilage biosynthesis in seeds, and trichome and root hair development (Szymanski *et al.*, 1998; Chen and Wang, 2019). To elucidate the function of *GL2* in cucumber, the putative full-length coding sequence (CDS) of Csa3M484840 (*CsHDIV10*) was cloned from cucumber inbred line 3461. Previously, Csa3M484840 was reported to have a CDS of 1428 bp (Fu *et al.*, 2013). In this study, we determined that the actual CDS of Csa3M484840 is 2346 bp and encodes a protein of 782 amino acid residues (see Supplementary Fig. S1). Protein domain analysis showed that Csa3M484840 contains the conserved homeodomain (HD), leucine zipper–loop–zipper (ZLZ) domain, StAR-related lipid-transfer (START) domain and the START-associated conserved domain (SAD) that are typical of HD-ZIP IV transcription factors (Yan *et al.*, 2017; Supplementary Fig. S2). Alignment and phylogenetic analysis showed that Csa3M484840 was clustered with *Gossypium hirsutum* GhHOX1 (NM_001327139.1), *G. hirsutum* GhHOX3 (XM_016828890.1) and Arabidopsis AtGL2 (AT1G79840), and it was therefore renamed CsGL2-LIKE (Supplementary Figs S2, S3). These observations indicated that CsGL2-LIKE belongs to the HD-ZIP IV subfamily and may be involved in epidermal cell determination.

### Overexpression of *CsGL2-LIKE* does not rescue the trichome phenotype of *gl2-8* mutants

*AtGL2* acts as the downstream gene of the MYB–bHLH–WD40 (MBW) transcriptional activator complex and initiates trichome formation in Arabidopsis. The *gl2-8* mutant showed abnormal trichome expansion with decreased trichome density in rosette leaves (Szymanski *et al.*, 1998; Shi *et al.*, 2012). To explore whether *CsGL2-LIKE* is functionally redundant to *AtGL2*, we transformed *CsGL2-LIKE* driven by the constitutive *Cauliflower mosaic virus* (CaMV) 35S promoter into the Arabidopsis *gl2-8* mutant. Overexpression of *AtGL2* in *gl2-8* mutant fully rescued the trichome differentiation defect, whereas ectopic expression of *35S:CsGL2-LIKE* in the *gl2-8* mutant was unable to rescue the *gl2-8* phenotype. These results suggest that there is functional divergence between *CsGL2-LIKE* and *AtGL2* (see Supplementary Fig. S4A–D).

### *CsGL2-LIKE* is highly expressed in cucumber anther

**Fig. 1.**
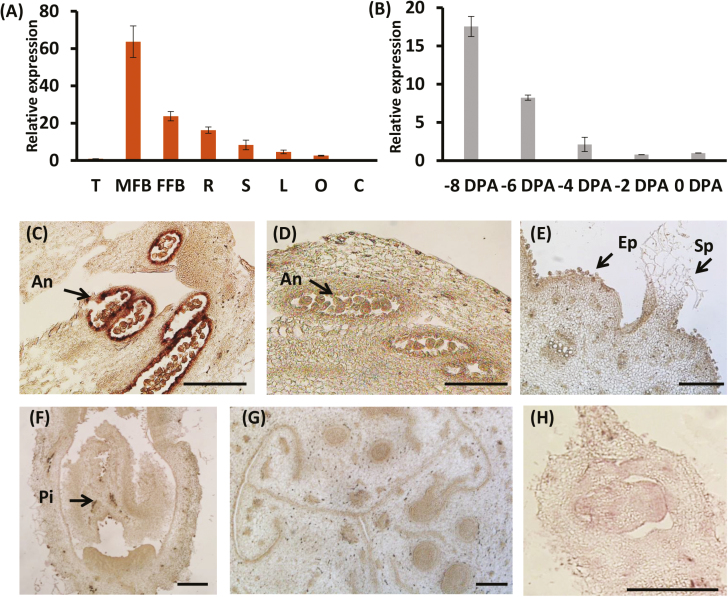
Identification of *CsGL2-LIKE* in cucumber. (A) qRT-PCR analysis of *CsGL2-LIKE* expression in various cucumber tissues and organs. C, cotyledon; FFB, female flower bud; L, leaf; MFB, male flower bud; O, ovary; R, root; S, stem; T, tendril. *Actin* (Csa6M484600) was used as an internal reference. (B) qRT-PCR analysis of *CsGL2-LIKE* expression in male flower bud from −8 DPA to 0 DPA. *Actin* (Csa6M484600) was used as an internal reference. The means ±SD of three independent biological samples are given. (C–H) A strong signal of *CsGL2-LIKE* was detected in the anthers of male flowers at −4 DPA. (D) A sense probe was used as a negative control. An, anther. (E–H) The signal was weak in the epidermal cells of the ovary at −4 DPA, female flowers at −4 DPA, locule at −4 DPA, and male flower buds at stage 2 (sepal primordia initiation; Bai *et al.*, 2004). Ep, epidermis; Sp, spine; Pi, pistil. Scale bars: 200 μm.

To better understand the biological function of *CsGL2-LIKE*, we evaluated its expression in various organs (roots, stems, leaves, female flower buds, male flower buds, ovary, cotyledon, and tendrils) using qRT-PCR. The highest expression of *CsGL2-LIKE* occurred in the male flower buds compared with other tissues (Fig. 1A). We then analysed *CsGL2-LIKE* transcript levels in the male flower buds from −8 to 0 DPA. The results indicate that the *CsGL2-LIKE* expression level gradually decreased with the growth of male flower buds (Fig. 1B). The expression patterns of *CsGL2-LIKE* at −4 DPA were further analysed by *in situ* hybridization experiments. *CsGL2-LIKE* expression was detected in the anthers (Fig. 1C, D), while no signal was detected in the ovary epidermal cells, female flower buds, and locules (Fig. 1E–G), which was consistent with the qRT-PCR results. Additionally, stage 2 (sepal primordia initiation; Bai *et al*., 2004) male flower buds showed no visible signal by mRNA *in situ* hybridization (Fig. 1H). Taken together, these observations suggest that *CsGL2-LIKE* may be involved in male flower development, particularly in the anthers.

### Silencing of *CsGL2-LIKE* delayed male flower blooming

**Fig. 2.**
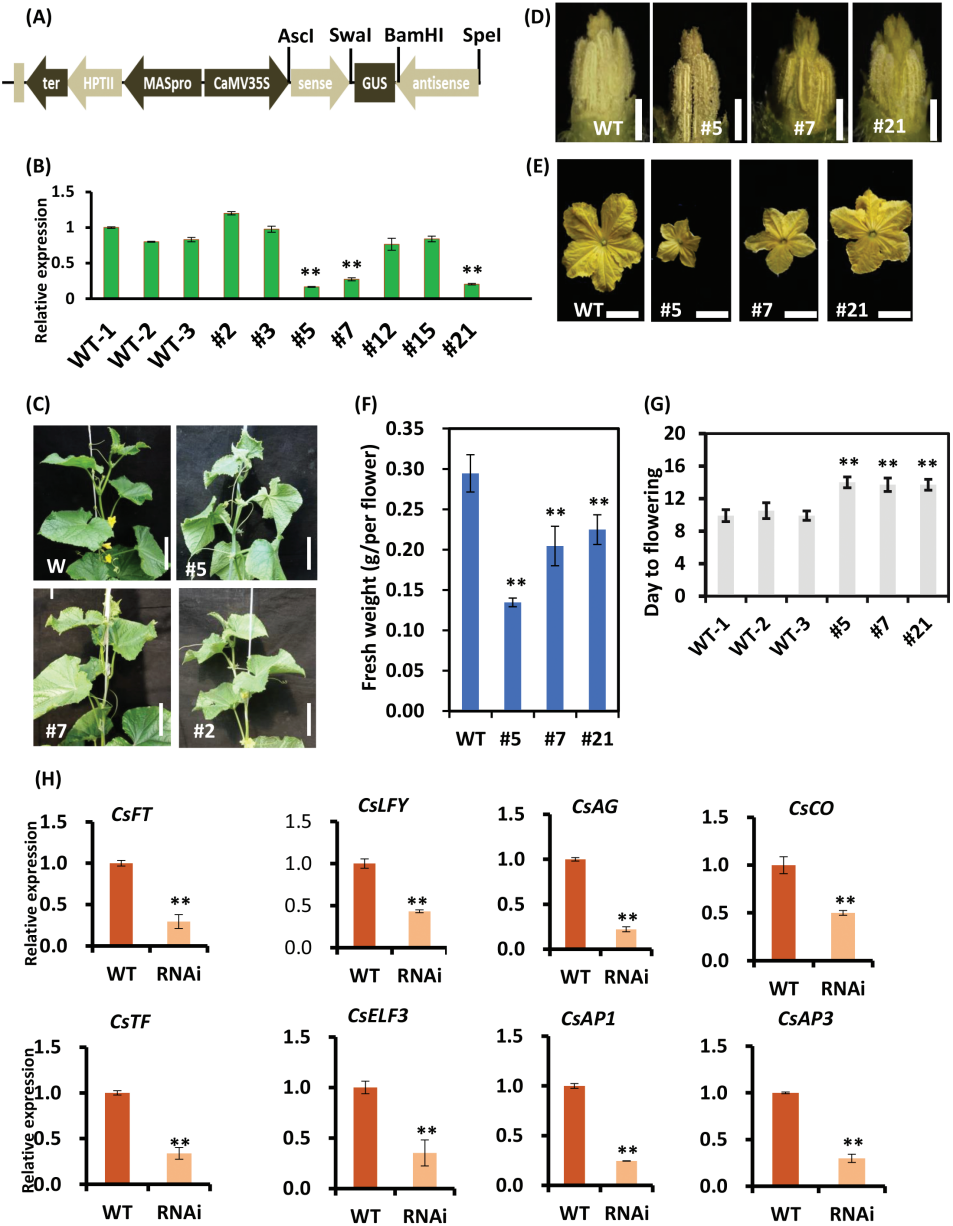
Silencing of *CsGL2-LIKE* delayed male flower blooming. (A) Schematic illustration of the CsGL2-LIKE RNAi expression vector used in this study. (B) The mRNA transcripts of *CsGL2-LIKE* in male flowers of *CsGL2-LIKE* RNA interference (RNAi) and wild-type plants at −6 DPA. *Actin* (Csa6M484600) was used as an internal reference. (C) The phenotype of wild-type and *CsGL2-LIKE* RNAi cucumber plants. Scale bar: 100 cm. (D) Stamens of wild-type and *CsGL2-LIKE* RNAi cucumber plants. Scale bar: 2 mm. (E) The male flower at 0 DPA of wild-type and *CsGL2-LIKE* RNAi cucumber plants. Scale bar: 500 mm. (F) The male flower fresh weight of wild-type and *CsGL2-LIKE* RNAi cucumber plants. Data are means ±SD (*n*=10; **P<0.01); (G) Compared with the wild-type plants, the *CsGL2-LIKE* RNAi plants exhibited delayed male flower blooming. The values shown are means ±SD (*n*=20). (H) Expression of ‘flowering time’ genes as determined by qRT-PCR. Data are means ±SD (*n*=3; **P<0.01).

To elucidate the function of *CsGL2-LIKE*, we constructed a *CsGL2-LIKE* RNA interference (RNAi) vector by using a 260 bp CDS fragment under the control of the CaMV35S promoter and introduced it into cucumber inbred line 3461 via an *Agrobacterium*-mediated cotyledon transformation method previously described by Clough and Bent (1998), Wang *et al.* (2014), and Liu *et al.* (2016) (Fig. 2A). Seven RNAi lines were obtained and three lines, 5, 7, and 21, which showed suppression in *CsGL2-LIKE* mRNA levels by 79%, 75%, and 78%, respectively, were selected for further analysis (Fig. 2B). The *CsGL2-LIKE* RNAi lines exhibited normal growth and development comparable to the WT plants (see Supplementary Fig. S5). However, at the time of flowering, differences in male flower development were observed between the *CsGL2-LIKE* RNAi lines and WT plants (Fig. 2C). All *CsGL2-LIKE* RNAi lines had smaller male flowers with lower fresh weight (Fig. 2D–F). Furthermore, the flowering time of male flowers was delayed in *CsGL2-LIKE* RNAi lines (Fig. 2G). To avoid any environmental errors, days to flowering was recorded during the spring and autumn growing seasons. In both seasons, male flowering times of *CsGL2-LIKE* RNAi plants were delayed (Supplementary Fig. S6). We then determined the expression levels of genes related to flowering time in the *CsGL2-LIKE* RNAi lines by qRT-PCR. Results showed that the expression levels of *FLOWERING LOCUS T* (*CsFT*), *LEAFY* (*CsLFY*), *AGAMOUS* (*CsAG*), *CONSTANS* (*CsCO*), *TERMINAL FLOWER* (*CsTF*), *EARLY FLOWERING 3* (*CsEFL3*), *APETALA1* (*CsAP1*), and *APETALA3* (*CsAP3*) were all down-regulated in the *CsGL2-LIKE* RNAi plants (Fig. 2H). These results indicate that *CsGL2-LIKE* may be a crucial regulator of male flower development and flowering time in cucumber.

### Silencing of *CsGL2-LIKE* also reduced pollen vigor and seed viability

**Fig. 3.**
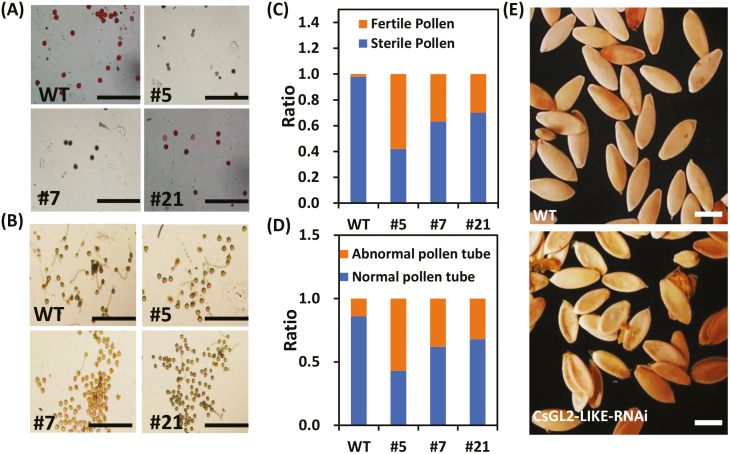
*CsGL2-LIKE* RNA interference (RNAi) plants exhibited a decrease in pollen vigor and maintained pollen tube extension. (A) Analysis of pollen vigor in wild-type and *CsGL2-LIKE* RNAi plants. Scale bar: 200 μm. (B) Analysis of pollen tube extension in wild-type and *CsGL2-LIKE* RNAi plants. Scale bar: 200 μm. (C) Ratio of sterile pollen to fertile pollen in wild-type and *CsGL2-LIKE* RNAi plants. (D) Ratio of normal to abnormal pollen tubes of wild-type and *CsGL2-LIKE* RNAi plants. (E) Seeds of wild-type and *CsGL2-LIKE* RNAi plants. Scale bar: 0.5 cm.

In addition to male flower development, the down-regulation of *CsGL2-LIKE* had noticeable effects on male fertility. Pollen vigor and pollen tube germination assays were conducted on male flowers at 0 DPA. The results revealed a decrease in pollen viability and disruption to pollen tube germination in all *CsGL2-LIKE* RNAi lines. Pollen grains from WT plants were bright red, healthy, and spherical in shape, whereas the *CsGL2-LIKE* RNAi lines contained irregularly shaped, non‐viable pollen grains (Fig. 3A, B). Additionally, several pollen tubes in *CsGL2-LIKE* RNAi lines failed to expand when treated with suitable medium, while most of the pollen tube in WT plants expanded. Complete male sterility was not observed in any of the *CsGL2-LIKE* RNAi lines tested for pollen viability. Fertility ranged from 42% in line 5 to about 70% in line 21, compared with WT plants, which averaged 98% fertile pollen (Fig. 3C). Likewise, *CsGL2-LIKE* RNAi plants had an average of 43% normal pollen tubes, while WT plants averaged 86% (Fig. 3D). Another significant effect of *CsGL2-LIKE* down-regulation was the inability of the RNAi lines to set seeds. Most of the seed capsules in the *CsGL2-LIKE* RNAi lines were empty post-fertilization, while normal seed formation was observed in WT plants (Fig. 3E). These results indicate that the down-regulation of *CsGL2-LIKE* not only delayed male flower development and reduced the number of male flowers, but also prevented pollen tube extension and decreased the pollen vigor and seed viability.

### Silencing of *CsGL2-LIKE* affected jasmonate-related genes

**Fig. 4.**
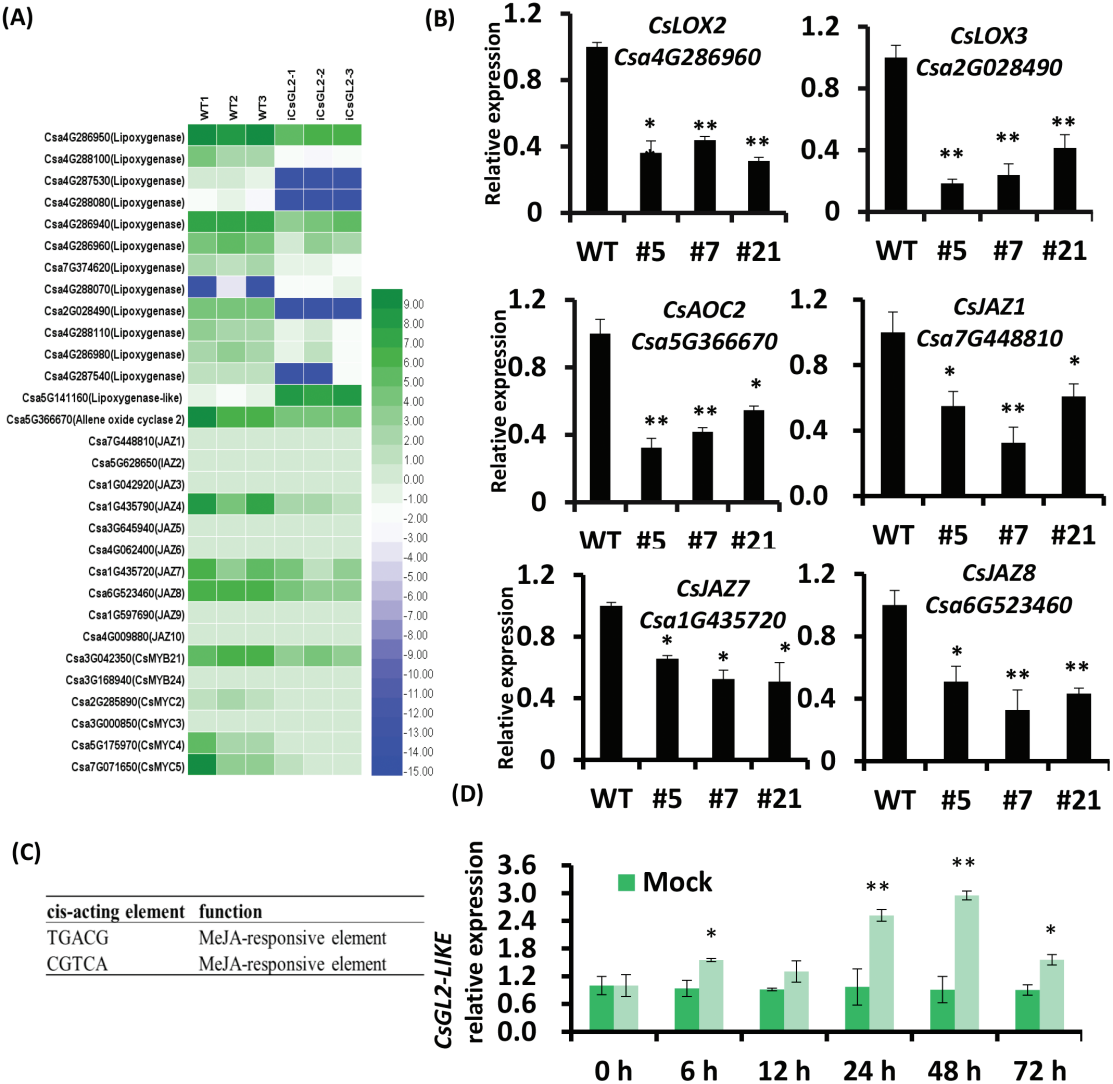
Silencing of *CsGL2-LIKE* affected jasmonate-related genes. (A) Heatmap representing differentially expressed genes associated with jasmonic acid metabolic process between wild type and *CsGL2-LIKE* RNA interference (RNAi) plants. (B) Relative expression of the genes involved in jasmonic acid metabolism. Data are means ±SD (*n*=3). (C) Identification of MeJA-responsive elements in the promoter region of *CsGL2-LIKE*. Promoter analysis and regulatory network prediction was accomplished by use of the PlantCARE program. (D) Relative expression of *CsGL2-LIKE* in 2-week-old cucumbers plants treated with 50 μM MeJA; 1% ethanol was used as a mock treatment. Leaf samples were taken every 6 h. *Actin* (Csa6M484600) served as an internal reference. Data are means ±SD (*n*=3; *P<0.05, **P<0.01, Student’s *t* test).

To further investigate the function of *CsGL2-LIKE* in male flower development, we performed a transcriptome analysis of *CsGL2-LIKE* RNAi and WT plants using RNA-Seq. A total of 3114 differentially expressed genes (DEGs) were identified between the *CsGL2-LIKE* RNAi and WT plants, including 1237 up-regulated and 1877 down-regulated genes (see Supplementary Fig. S7A). Gene ontology (GO) enrichment analysis indicated that most DEGs were involved in plant hormone signal transduction, hormone signaling pathway, response to organic substance, and pollen development (Supplementary Fig. S7B). Interestingly, we observed that several JA-signal-related genes were differently expressed in the *CsGL2-LIKE* RNAi plants (Fig. 4A; Supplementary Table S3). JA is known to play a significant role in regulating anther development and flowering time (Song *et al.*, 2011; Qi *et al.*, 2015; Zhai *et al.*, 2015). The qRT-PCR analysis revealed that the expression of other JA-related genes, *CsLOX2*, *CsLOX3*, *CsAOC2*, *CsJAZ1*, *CsJAZ7*, and *CsJAZ8*, were down-regulated in the *CsGL2-LIKE* RNAi plants (Fig. 4B). We also observed that Csa2G080170, a homolog of Arabidopsis *AtMYC2*, was also down-regulated in the *CsGL2-LIKE* RNAi plants (Supplementary Fig. S7D). AtMYC2 is a key component of the JA signaling pathway and is known to regulate stamen development and flowering time (Fernández-Calvo *et al.*, 2011; Schweizer *et al.*, 2013; Qi *et al.*, 2015). Promoter analysis revealed several putative MeJA responsive *cis*-acting elements in the promoter region of *CsGL2-LIKE* (Fig. 4C). To determine the effect of MeJA on *CsGL2-LIKE*, we applied MeJA treatments to 2-week-old cucumber plants. *CsGL2-LIKE* expression was significantly up-regulated from 0 to 72 h in MeJA-treated plants, while no significant change in expression was observed in control plants (Fig. 4D). Silencing of *CsGL2-LIKE* also affected the expression of several transcription factors including the AP2/ERF transcription factors and a zinc finger transcription factor (Supplementary Fig. S7C). Several PHD finger proteins, including MALE STERILITY 1 (Ms-1), which controls male fertility in cucumber, were also down-regulated in *CsGL2-LIKE* RNAi lines (Zhang *et al.*, 1994; Supplementary Fig. S7D). Similarly, several genes involved in pollen and pollen tube development were also down-regulated (Supplementary Table S4). Taken together, we speculated that *CsGL2-LIKE* might interact with jasmonate-related genes to regulate male flower development in cucumber.

### CsGL2-LIKE interacts with a subset of CsJAZ proteins

**Fig. 5.**
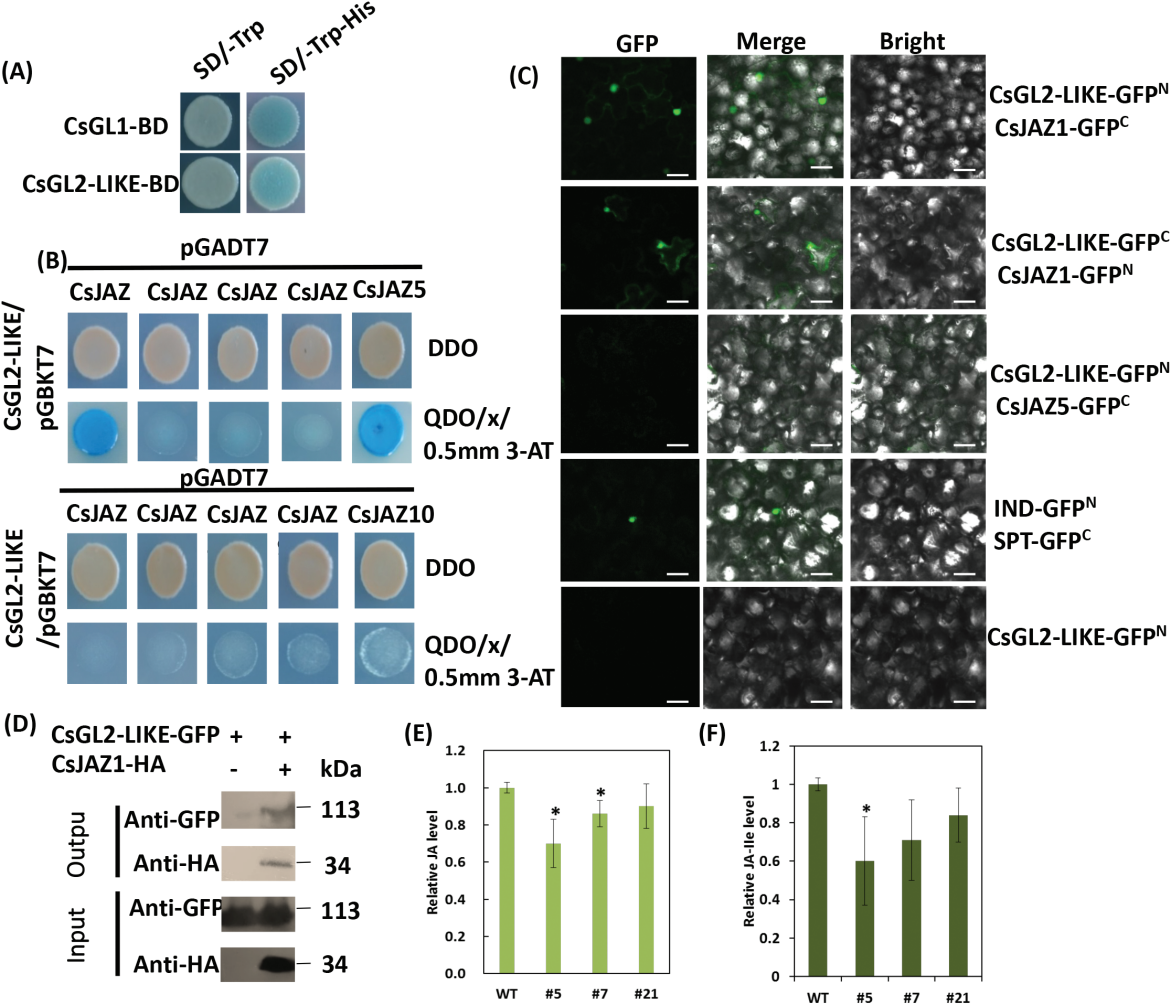
CsGL2-LIKE interacts with the jasmonate (JA) ZIM protein CsJAZ1. (A) Transactivation analysis of CsGL2-LIKE in yeast strain AH109 grown in SD−Trp and SD−Trp/−His/X-α-Gal media. (B) CsGL2-LIKE interacts with CsJAZ1 and CsJAZ5 in yeast. (C) Detection of CsGL2-LIKE/CsJAZ1 and CsGL2-LIKE/CsJAZ5 interactions by bimolecular fluorescence complementation assay in *N. benthamiana* leaves. IND and SPT were used as positive control and CsGL2-LIKE-GFP^N^ was used as negative control. Scale bar: 50 μm. (D) Detection of the CsGL2-LIKE–GFP interaction by co-immunoprecipitation analysis. GFP-tagged CsGL2-LIKE and HA-tagged CsJAZ1 were expressed in *N. benthamiana* leaves for the analysis. (E, F) Measurement of endogenous JA and JA-Ile content in male flower of wild-type and CsGL2-LIKE-RNAi plants. Data are means ±SD (*n*=3; *P<0.05, Student’s *t* test).

JAZ proteins are central components of the JA-signaling pathway and modulate several floral development-related TFs, including MYC2/3/4/5, TOE1/2, MYB21, and MYB24 (Cheng *et al*, 2011; Fernández-Calvo *et al.*, 2011; Song *et al.*, 2011; Schweizer *et al.*, 2013; Boter *et al.*, 2015; Qi *et al.*, 2015; Zhai *et al.*, 2015; Wang *et al.*, 2017). Transcriptome and qRT-PCR analyses revealed that the expression of several *CsJAZ* genes was down-regulated in the *CsGL2-LIKE* RNAi lines. To further dissect the function of CsGL2-LIKE and the molecular mechanism regulating male flower development, yeast two-hybrid (Y2H) assays were used to screen interaction partners of CsGL2-LIKE in the cucumber genome. The yeast transformation of CsGL2-LIKE protein showed weak transcriptional activation activity (Fig. 5A), which was similar to that of CsGL1 reported by Li *et al.* (2015). Nine putative interacting proteins were obtained, which included JAZ protein h (CsJAZ1; Csa7G448810), a homolog of AtJAZ1 (AT1G19180) (see Supplementary Table S5). Additional Y2H assays indicated that CsGL2-LIKE interacted directly with CsJAZ1 and CsJAZ5 (Csa3G645940; Fig. 5B). In further bimolecular fluorescence complementation (BiFC) and co-immunoprecipitation (Co-IP) assays, the interaction between CsGL2-LIKE and CsJAZ1 was proven *in planta* (Fig. 5C, D). However, no interaction was observed between CsGL2-LIKE and CsJAZ5. The spatial expression pattern of *CsJAZ5* was also different from that of *CsGL2-LIKE*, suggesting that *CsJAZ5* may play a different role in cucumber (Supplementary Fig. S8). We also observed that endogenous JA levels in male flowers decreased in *CsGL2-LIKE* RNAi lines 5, 7, and 21 by 30%, 14%, and 10%, respectively, while endogenous JA-Ile levels decreased 40%, 29%, and 16%, respectively (Fig. 5E, F). These data support the existence of direct interactions between CsGL2-LIKE and CsJAZ1. We speculate that JA signals modulate CsGL2-LIKE to regulate male flower development in cucumber.

### CsJAZ1 is highly expressed in the anthers

**Fig. 6.**
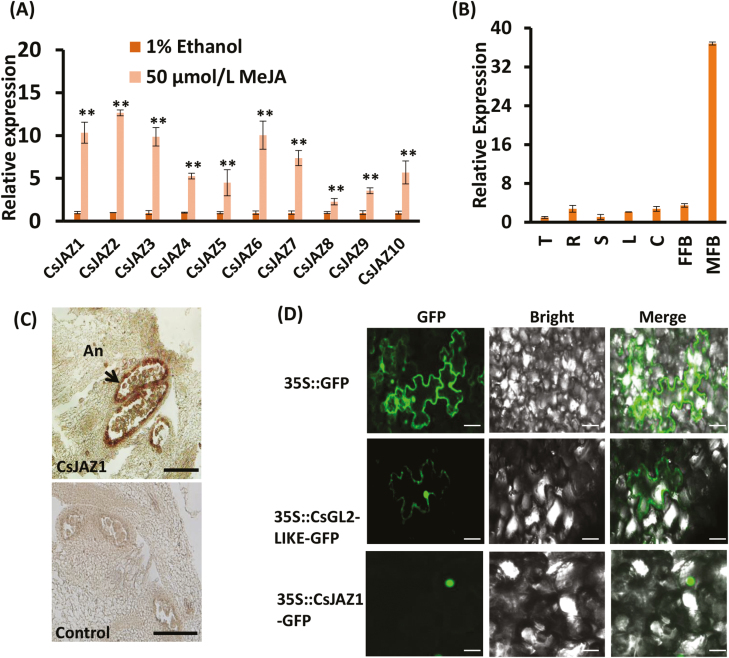
*CsJAZ1* is highly expressed in the anthers. (A) qRT-PCR analysis of *CsJAZ* genes in cucumber in response to MeJA treatment. Data are means ±SD (*n*=3; **P<0.01, Student’s *t* test). (B) qRT-PCR analysis of the expression of *CsJAZ1* in various cucumber organs. C, cotyledon; FFB, female flower bud; L, leaf; MFB, male flower bud; R, root; S, stem; T, tendril. Data are means ±SD (*n*=3). *Actin* (Csa6M484600) was used as an internal reference. (C) *In situ* hybridization analysis of *CsJAZ1* in cucumber anthers. An, anther. Scale bar: 200 μm. (D) Subcellular localization of 35S::CsGL2-LIKE-GFP and 35S::CsJAZ1-GFP in green fluorescence, merged, and bright field channels. The expression of 35S::GFP in *Nicotiana benthamiana* was used as a positive control. Scale bar: 50 μm.

Based on sequence alignments and phylogeny analysis, all CsJAZ proteins were closely related and contained the highly conserved ZIM and Jas domains (see Supplementary Figs S9, S10). Upon MeJA treatment, all *CsJAZ* genes were up-regulated (Fig. 6A), indicating that CsJAZs may play a similar role to other plant JAZs, and acts as a repressor in the JA signaling pathway in cucumber. qRT-PCR analysis revealed that *CsJAZ1* was highly expressed in male flower buds at −4 DPA in line 3461 (Fig. 6B). Similar results were obtained by *in situ* hybridization experiments, indicating that *CsJAZ1* functioned in anthers (Fig. 6C), showing overlapping expression with *CsGL2-LIKE*. Transient expression of CsGL2-LIKE–GFP fusion protein in *N. benthamiana* leaves showed that CsGL2-LIKE localizes in cytosol and nucleus (Fig. 6D). Similarly, CsJAZ1, which is degraded by the SCF^COI1^ complex (Chini *et al.*, 2007), localizes in the nucleus. These results suggested that CsJAZ1 plays a role in the regulation of male flower development in cucumber.

## Discussion

Cucumber is a well-established model system for unisexual flower development (Bai and Xu, 2013; Boualem *et al.*, 2015). However, knowledge regarding the genetic and molecular mechanisms controlling cucumber male flower development is limited. Flowering is reported to be regulated by several TFs and hormone signals (Szymanski *et al.*, 1998; Vernoud *et al.*, 2009; Shi *et al.*, 2012; Fu *et al.*, 2013). In this study, we demonstrate that CsGL2-LIKE, a member of the cucumber HD-ZIP IV subfamily, and CsJAZ1, belonging to the jasmonate ZIM-domain family of transcriptional repressors, forms a complex that regulates male flower development. Furthermore, the silencing of *CsGL2-LIKE* caused partial male sterility and delayed male flowering by repressing *FT*. Our findings give new insights into the role of HD-ZIP IV proteins in male flower development and provide effective gene resources for improving cucumber productivity.

### CsGL2-LIKE is a HD-ZIP IV transcription factor whose transcripts accumulate in anther

GL2 is a homeodomain transcription factor that is known to promote trichome initiation in shoots and mucilage biosynthesis in seeds, and to inhibit hair formation in roots (Rerie *et al.*, 1994; Chen and Wang, 2019). Here, we studied the function of *CsGL2-LIKE* in cucumber. Protein sequence alignment revealed that CsGL2-LIKE shares high sequence identity with homologs AtGL2 and GhHOX1, and contains the conserved HD, ZLZ, START, and HD-SAD domains that are typical of HD-ZIP IV TFs (see Supplementary Fig. S2). Several reports indicate that *AtGL2* acts as a key gene promoting trichome initiation in Arabidopsis (Szymanski *et al.*, 1998; Chen and Wang, 2019). Here, overexpression of *AtGL2* in the *gl2-8* mutant fully rescued the trichome differentiation defect, whereas the ectopic expression of *CsGL2-LIKE* in the *gl2-8* mutant was unable to rescue the *gl2-8* phenotype (Supplementary Fig. S4). Furthermore, *CsGL2-LIKE* expression was low in cucumber epidermal cell, and down-regulation of *CsGL2-LIKE* did not influence trichome initiation in cucumber. These results indicate that *CsGL2-LIKE* may not play a role in trichome initiation, suggesting functional divergence between the homologous genes. Vernoud *et al.* (2009) and Depège-Fargeix *et al.* (2011) reported that the mRNA transcript of several HD-ZIP IV TFs accumulated in pollen grains and tapetum, suggesting a role in male fertility. Likewise, the *CsGL2-LIKE* transcript predominantly accumulated in male flower anther (Fig. 1). These observations suggest that *CsGL2-LIKE*, although similar in structure to other *GL2* homologs, may play a role in male flower development in cucumber.

### *CsGL2-LIKE* is involved in the regulation of male flowering and fertility in cucumber

Several homeodomain proteins have been shown to regulate flowering time and male sterility. The GL2-type homeobox mutant *Roc4* is known to promote flowering time in rice (Wei *et al.*, 2016). Likewise, the maize HD‐ZIP IV gene *outer cell layer 4* (*OCL4*) has been reported to affect male sterility, while ectopic expression of maize *OCL1* in rice delays flowering time (Vernoud *et al.*, 2009; Depège-Fargeix *et al.*, 2011). In this study, we provide evidence that demonstrates the role of *CsGL2-LIKE* in male flower development. Expression analysis revealed that *CsGL2-LIKE* transcript accumulated mainly in the male flower buds compared with other tissues, and gradually declined with the growth of male flower buds (Fig. 1A, B). Further, *in situ* hybridization experiments confirmed that *CsGL2-LIKE* was expressed in the anthers of male flower buds (Fig. 1C, D). *CsGL2-LIKE* expression pattern was like that of *OCL4*, with transcripts accumulating in the gynoecium, stamens, anthers, and young male maize flowers (Vernoud *et al.*, 2009). These data indicate that *CsGL2-LIKE* plays a role in regulating male flower development.

The silencing of *CsGL2-LIKE* resulted in delayed male flowering and a decrease in male flower size and weight (Fig. 2D–G). Additionally, a decrease in pollen viability and disruption to pollen tube germination were observed in all *CsGL2-LIKE* RNAi lines (Fig. 3A–D). Vernoud *et al.* (2009) reported similar findings in maize where *ocl4‐1* and *ocl4‐2* mutants showed varying degrees of male sterility. The *ocl4‐1* and *ocl4‐2* mutant anthers contained irregularly shaped, non‐viable pollen grains that were similar to the pollen in the *CsGL2-LIKE* RNAi lines. Another significant effect of *CsGL2-LIKE* down-regulation was the inability of the RNAi lines to set seeds. Most of the seed capsules in the *CsGL2-LIKE* RNAi lines were empty post-fertilization, while normal seed formation was observed in the wild type plants (Fig. 3E). It is known that *CsFT* controls early flowering in cucumber (Lu *et al.*, 2014). Transcriptome and qRT-PCR analyses reveal that floral integrator genes *CsFT*, *CsLFY*, and other flowering-related genes were down-regulated in *CsGL2-LIKE* RNAi plants (Fig. 2H). Taken together, our data identified *CsGL2-LIKE* as a positive regulator of male flowering and fertility in cucumber.

### CsGL2-LIKE interacts with CsJAZ1 to form a HD-ZIP IV–CsJAZ1 complex and might be involved in male flower development

JAs are signaling molecules that regulate a wide range of developmental processes. The accumulation of JA-Ile causes the ubiquitination and degradation of the COI1–JAZ complex leading to the expression of several early JA-responsive genes (Wasternack and Hause, 2013; Chini *et al.*, 2016), whereas the absence of JA-Ile causes JAZs, along with several associated co-repressors, to bind and block TFs that target early JA-responsive genes. Therefore, JAZs act as negative regulators of the JA signaling pathways (Chini *et al.*, 2007). In this study, analysis of the promoter region of *CsGL2-LIKE* revealed several putative MeJA responsive *cis*-acting elements, indicating a relationship between *CsGL2-LIKE* and JA. Furthermore, Y2H library screening assays showed that CsGL2-LIKE interacts directly with nine putative proteins, including a JAZ protein, CsJAZ1 (see Supplementary Table S5). Previous studies have shown that JAZ proteins can repressed JA-regulated male flowering and affect fertility. Thines *et al.* (2007) and Chung and Howe (2009) reported that the overexpression of a truncated form of JAZ1 or an alternatively spliced form JAZ10.4, lacking the Jas domain, resulted in male sterility, whereas Oblessuc *et al.* (2020) reported that the overexpression of a dominant-negative form of *JAZ1ΔJas* results in early flowering. In further BiFC and Co-IP assays, CsGL2-LIKE interacts directly with CsJAZ1, indicating that CsJAZ1 may participate in the regulation of CsGL2-LIKE (Fig. 5C, D). Likewise, other JAZ proteins have been reported to interact with the R2R3-MYB TFs to mediate male fertility in Arabidopsis (Song *et al.*, 2011), while JA-mediated stamen development and seed production are also regulated by a JAZ–bHLH–MYB complex (Qi *et al.*, 2015).

CsJAZ1 was predominantly expressed in the male flower buds, showing overlapping expression with CsGL2-LIKE (Fig. 6B, C). Furthermore, the level of endogenous JA and JA-Ile was lower in *CsGL2-LIKE* RNAi plants than in WT plants (Fig. 5E, F), and application of exogenous MeJA to the *CsGL2-LIKE* RNAi plants did not rescue the male flowering time phenotype of WT plants. Interestingly, a cluster of ethylene-related genes was down-regulated in *CsGL2-LIKE* RNAi plants, indicating that ethylene may also be involved in regulating male flower development in cucumber (see Supplementary Fig S7C, D). These data confirm the relationship between CsGL2-LIKE and CsJAZ1. Using reverse genetic methods, our results uncover a new role for the HD-ZIP IV–JAZ complex in regulating male flowering and fertility. Future studies to obtain knockout transgenic lines of CsGL2-LIKE and CsJAZ1 by using CRISPR/Cas9 technology would help research into the molecular mechanism of male flowering and fertility in cucumber.

## Supplementary data

Supplementary data are available at *JXB* online.

Fig. S1. Cloning of the *CsGL2-LIKE* coding DNA sequence (CDS).

Fig. S2. Structural analysis of *CsGL2-LIKE*.

Fig. S3. Phylogenetic tree analysis of CsGL2-LIKE.

Fig. S4. The trichome numbers did not differ between the *gl2-8* mutant and *35S::CsGL2-LIKE::gl2-8* transgenic line.

Fig. S5. Fruit morphology, leaf morphology, plant height, and stem diameter in wild-type and *CsGL2-LIKE* RNAi plants were similar.

Fig. S6. *CsGL2-LIKE* RNA interference (RNAi) plants exhibited delayed male flowering in autumn 2017 and spring 2018.

Fig. S7. Transcription profiling of *CsGL2-LIKE* RNAi transgenic plants.

Fig. S8. Quantitative real-time PCR (qRT-PCR) analysis of CsJAZ5 (Csa3G645940).

Fig. S9. Sequence alignment of 10 JAZs in cucumber.

Fig. S10. Phylogenetic tree analysis of 10 JAZs in cucumber.

Table S1. Primers used in this study.

Table S2. Gene information used in this study.

Table S3. List of genes that were significantly down/up-regulated in the GL2-like RNAi as compared with the WT plant.

Table S4. Genes involved in pollen and pollen tube development in DEGs of CsGL2-LIKE RNAi plants.

Table S5. List of proteins that interacted with CsGL2-LIKE.

eraa251_suppl_Suplementary_Figures-S1-S10_Tables-S1-S4

eraa251_suppl_Suplementary_Tables-S5
