# Supplementary material for: The HD-ZIP IV transcription factor GL2-LIKE regulates male flowering time and fertility in cucumber
Source: J Exp Bot. 2020 Jun 3;71(18):5425–37. doi: 10.1093/jxb/eraa251 (PMC7501822; doi:10.1093/jxb/eraa251)
Supplement: eraa251_suppl_Suplementary_Figures-S1-S10_Tables-S1-S4 [file eraa251_suppl_suplementary_figures-s1-s10_tables-s1-s4.pdf]

Supplemental Figure S1. Cloning of the *CsGL2-LIKE* coding DNA sequence (CDs).

The length of the *CsGL2-LIKE* gene is 2346 bp. The complete coding DNA sequence (CDs) consists of two predicted genes: *Csa3M484840* and *Csa3M484830*.

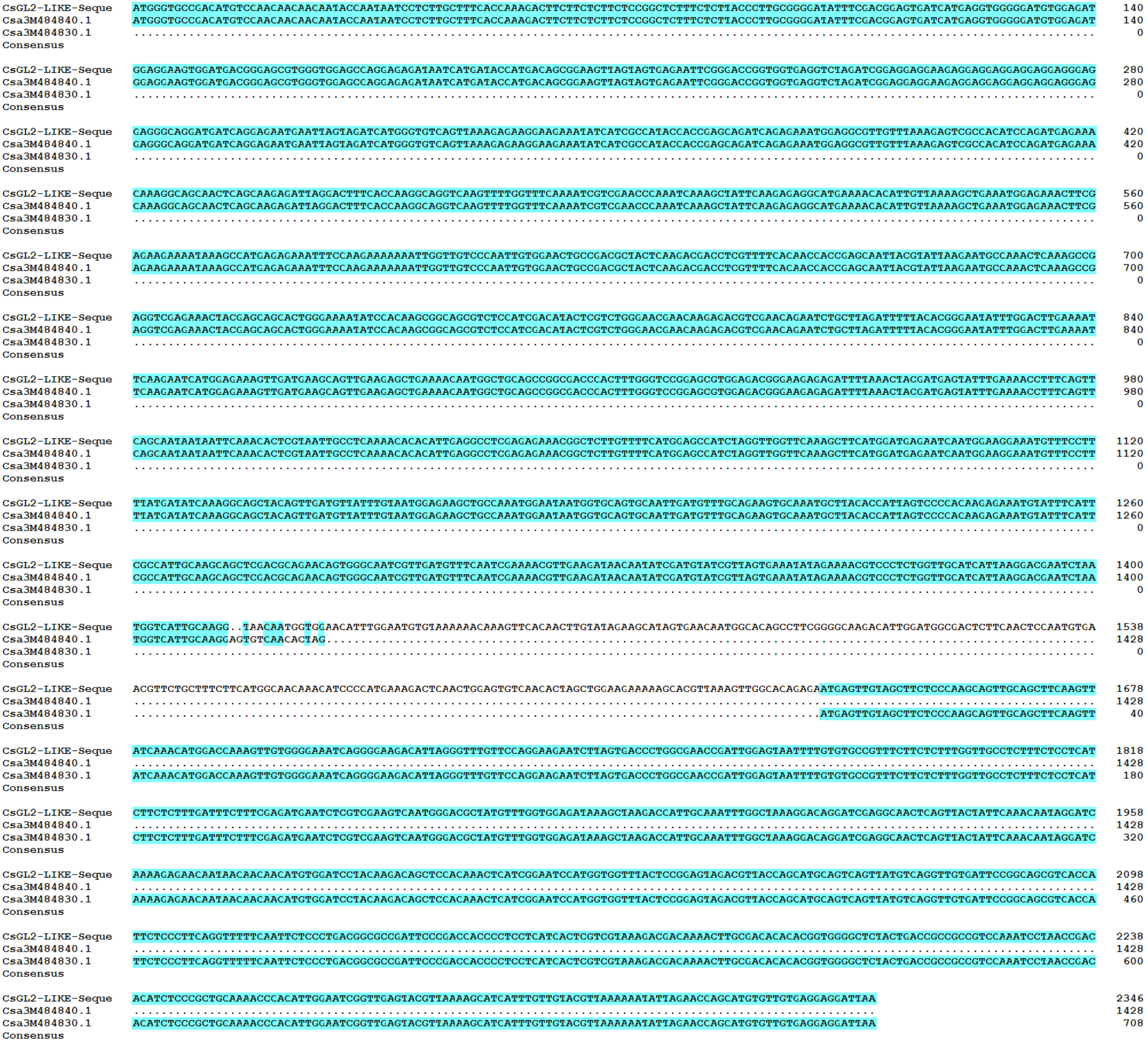

Supplemental Figure S2. Structure analysis of *CsGL2-LIKE*.

Protein sequence alignment of CsGL2-LIKE and homologs from *Arabidopsis thaliana* AtGL2 (AT1G79840) and *Gossypium hirsutum* GhHOX1 (NM_001327139.1) were analyzed by using MEGA 5.0 and BoxShade (https://embnet.vital-it.ch/software/BOX_form.html).The red line indicates the homeodomain (HD), the green line indicates Leucine-ZIP like domain, the blue line indicates the StAR-related lipid-transfer (START) domain and the yellow line indicates the START-associated conserved domain (SAD) domain.

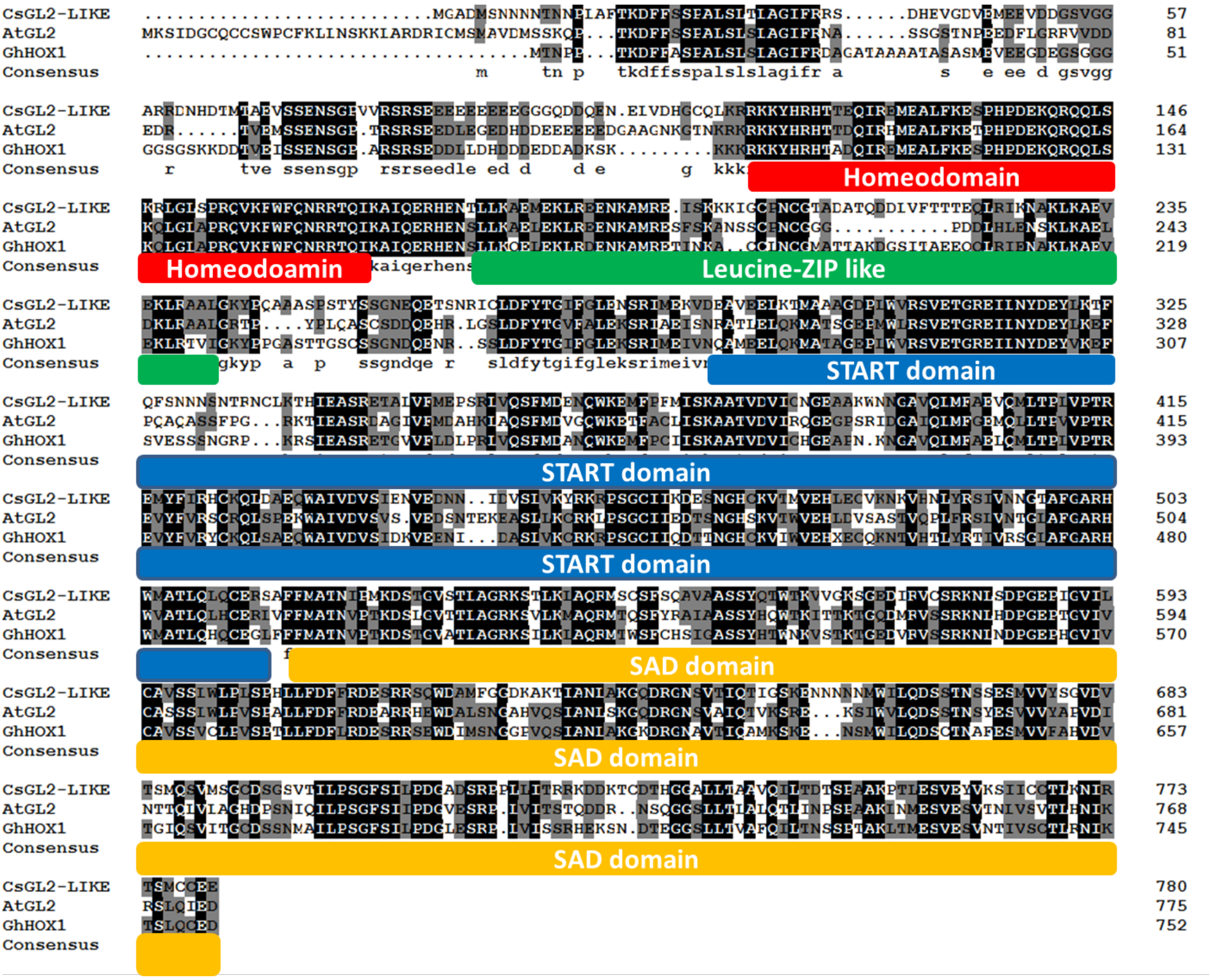

Supplemental Figure S3. Phylogenetic tree analysis of CsGL2-LIKE.

The neighbor-joining phylogenetic tree with Poisson correction is based on amino acid sequence alignments. The phylogenetic tree was constructed using MEGA 5. Bootstrap analysis was employed using 5000 replicates. CsGL2-LIKE is marked with ▲. The HD-ZIP IVs GenBank accession numbers are listed in Supplemental Table S4.

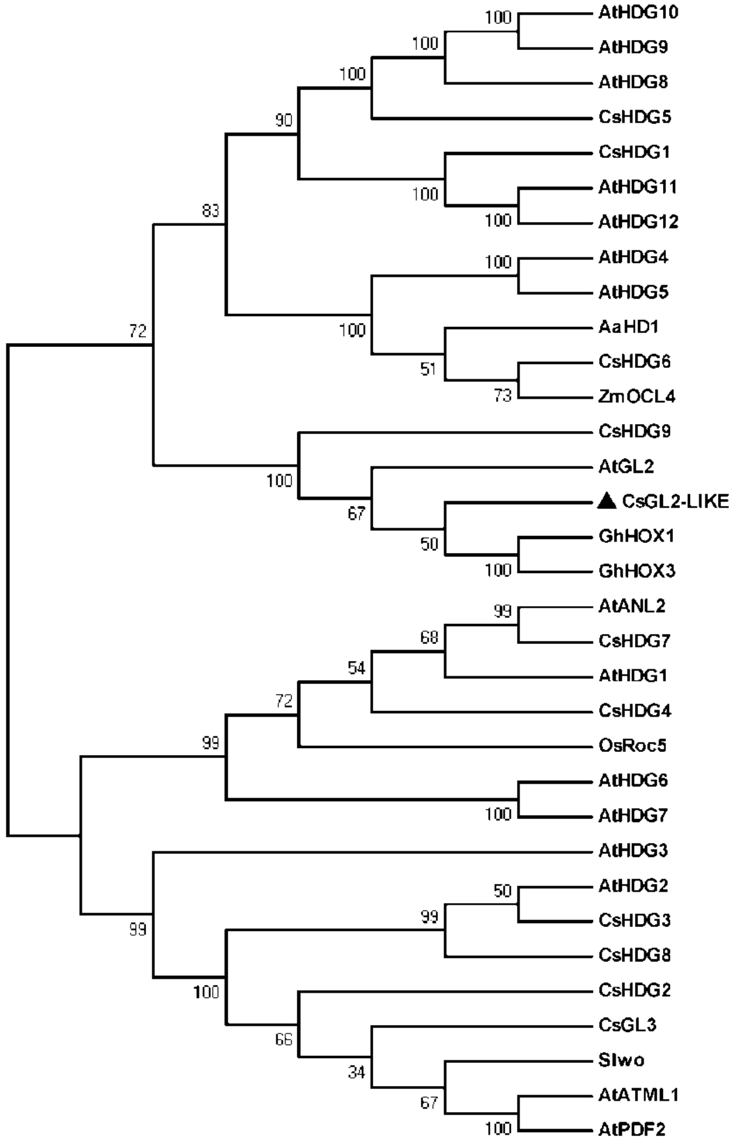

Supplemental Figure S4. The trichomes numbers did not differ between the *gl2-8* mutant and *35S::CsGL2-LIKE::gl2-8* transgenic line.

(A) Quantitative real-time PCR (qRT-PCR) analysis of *CsGL2-LIKE* expression in *gl2-8* mutant. #1, #2 and #3 were *gl2-8* mutant. G-1 to G-5 was *CsGL2-LIKE* ectopic expression in *gl2-8* mutant T2 lines. Actin2 (AT2G32700) was used as an internal reference. **(B)** Phenotype of *gl2-8, 35S:: CsGL2-LIKE::gl2-8* transgenic lines and *35S:: AtGL2::gl2-8* transgenic lines. Bar=200 mm. **(C)** Trichome number in *gl2-8* mutant and *35S::CsGL2-LIKE::gl2-8* transgenic lines. #1, #2 and #3 were *gl2-8* mutant. G-3, G-4 and G-5 were CsGL2-LIKE ectopic expression of *gl2-8* mutant lines. **(D)** Western blot detected the protein of CsGL2-LIKE in Arabidopsis wild-type and *35S::CsGL2-LIKE::gl2-8* transgenic line. GFP protein was used as positive control and mutant *g2l-8* was used as negative control. The means ± standard deviations (SDs) of three independent biological samples (each with a technical replicate) are given (*, P<0.05; **, P<0.01).

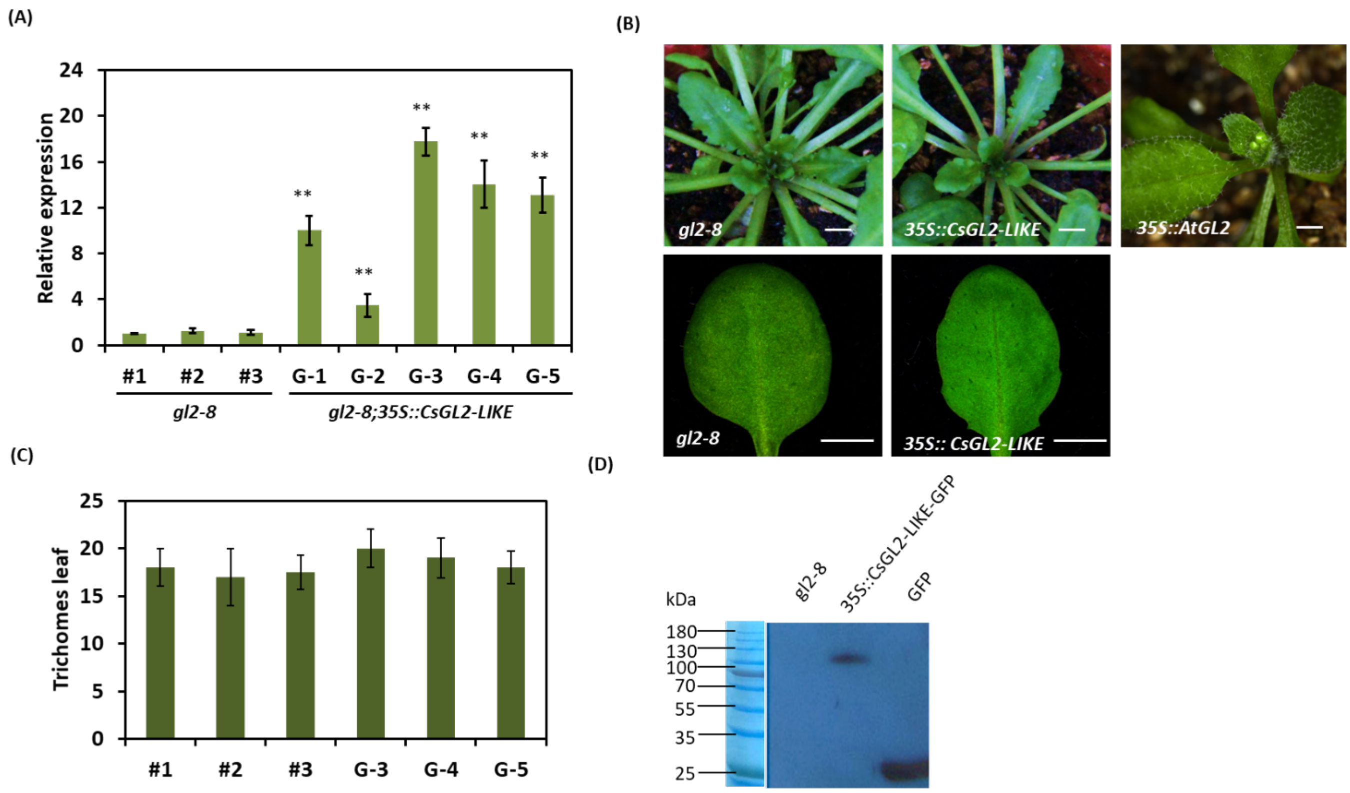

Fig S4.

Supplemental Figure S5. Fruit morphology, leave morphology, plant height and stem diameter in wild-type and *CsGL2-LIKE* RNAi plants were similar.

(A-B) Cucumber fruit at 12 DPA in wild-type and *CsGL2-LIKE* RNAi plant. Bar=6cm. (C) Leaves in wild-type and *CsGL2-LIKE* RNAi plant. Bar=6cm. (D) Table showed the plant height, stem width, leaf area and fruit weight were not significantly different in wild-type and *CsGL2-LIKE* RNAi plantsa.

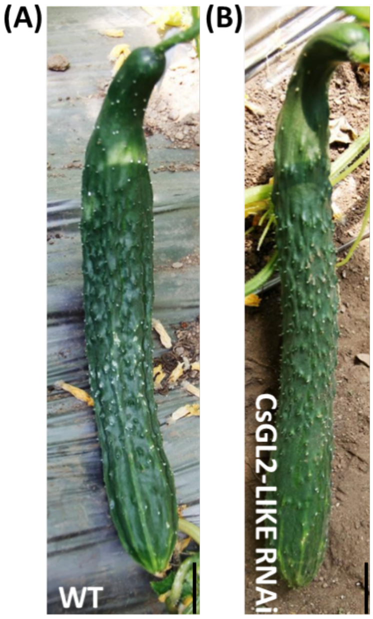

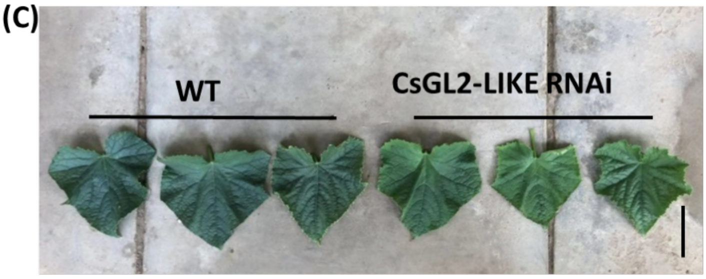

(D)

Phenotype	WT	CsGL2-LIKE RNAi
Plant height(m)	1.72±0.15a	1.70±0.23a
Stem width(cm)	1.02±0.08a	0.98±0.04a
Leave area(cm^2)	101.2±6.46a	94.5±3.42a
Fruit weight(g)	145.3±4.45a	147.8±1.43a

Supplemental Figure S6. ***CsGL2-LIKE*** **RNA interference (RNAi) plants exhibited delayed male flowering in spring 2018 and autumn 2017.**

(A-B) Days to flowering for male flowers in spring 2018 and autumn 2017 for both wild-type and *CsGL2-LIKE* RNA interference (RNAi) plants. Data are means ± SD of ten independent biological samples (each with a technical replicate) are given.

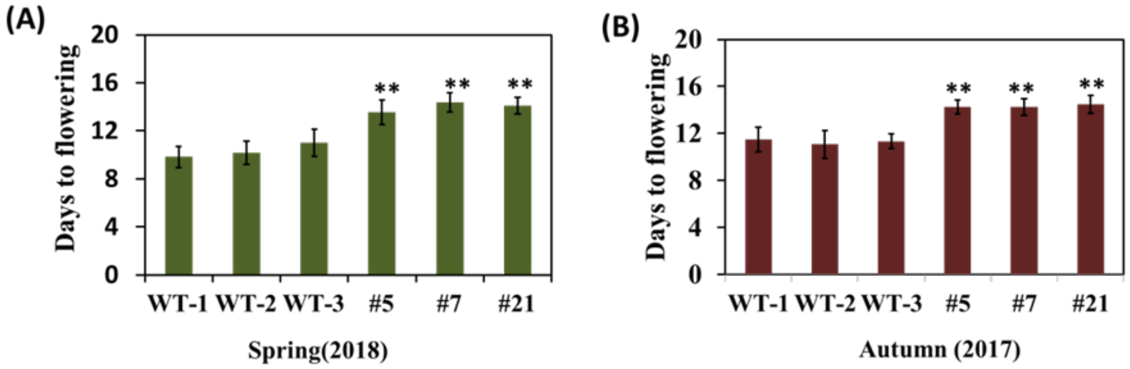

Supplemental Figure S7. Transcription profiling of *CsGL2-LIKE* RNAi transgenic plants.

(A) Venn diagram showed the number of DEGs in *CsGL2-LIKE* RNAi transgenic plants. (B) GO enrichment showed that top 10 gene ontology go terms. (C) Function categories of transcription factors upregulated in *CsGL2-LIKE* RNAi transgenic plants. (D) Heat map profiled of selected transcription factors.

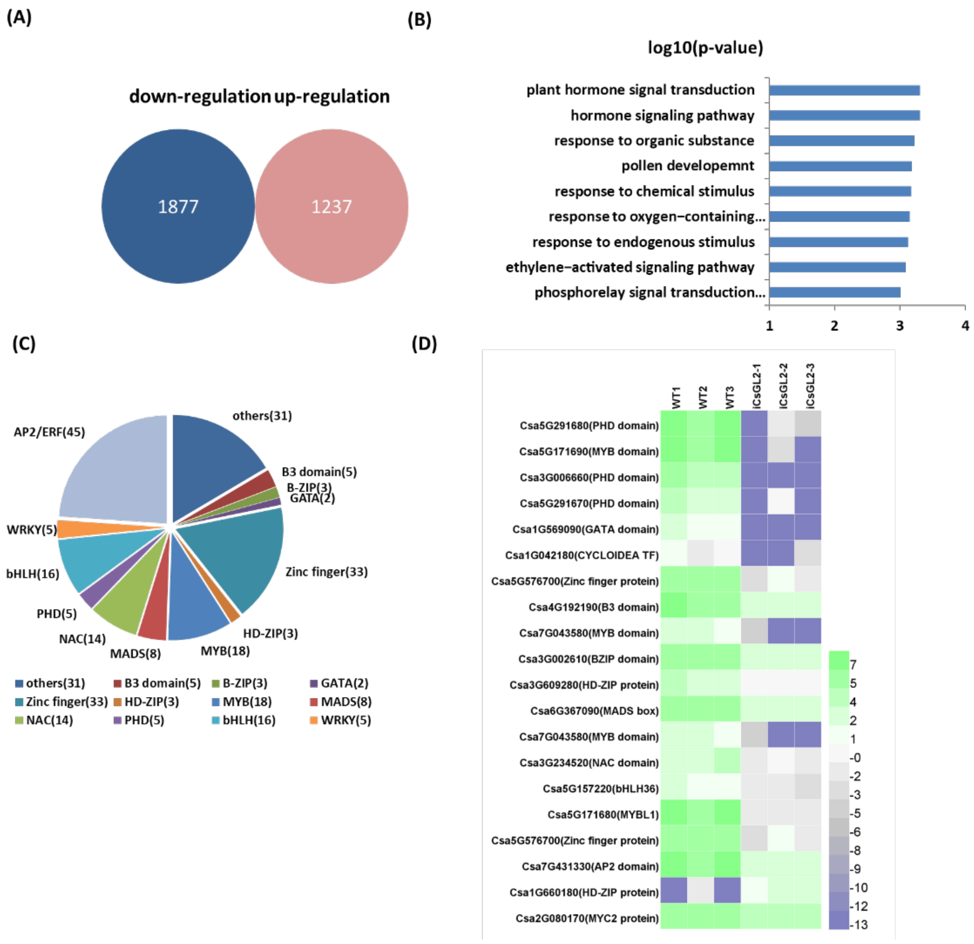

Supplemental Figure S8. Quantitative real-time PCR (qRT-PCR) analysis of CsJAZ5 (Csa3G645940). qRT-PCR analysis of CsJAZ5 in different cucumber tissues. L, leaf; MF, male flower; FF, female flower; R, root; O, ovary; S, stem. Actin (Csa6M484600) was used as an internal reference.

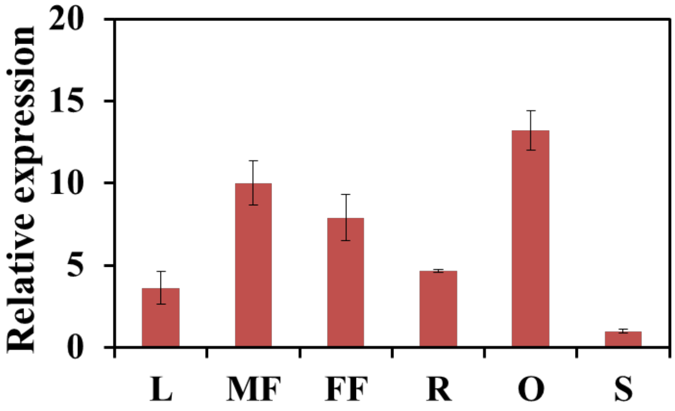

Supplemental Figure S9. Sequence alignment of ten JAZs in cucumber.

The amino acid sequence alignment of ten cucumber JAZ proteins. The conserved ZIM domain is indicated in the red box, and the Jas domain is indicated in the blue box.

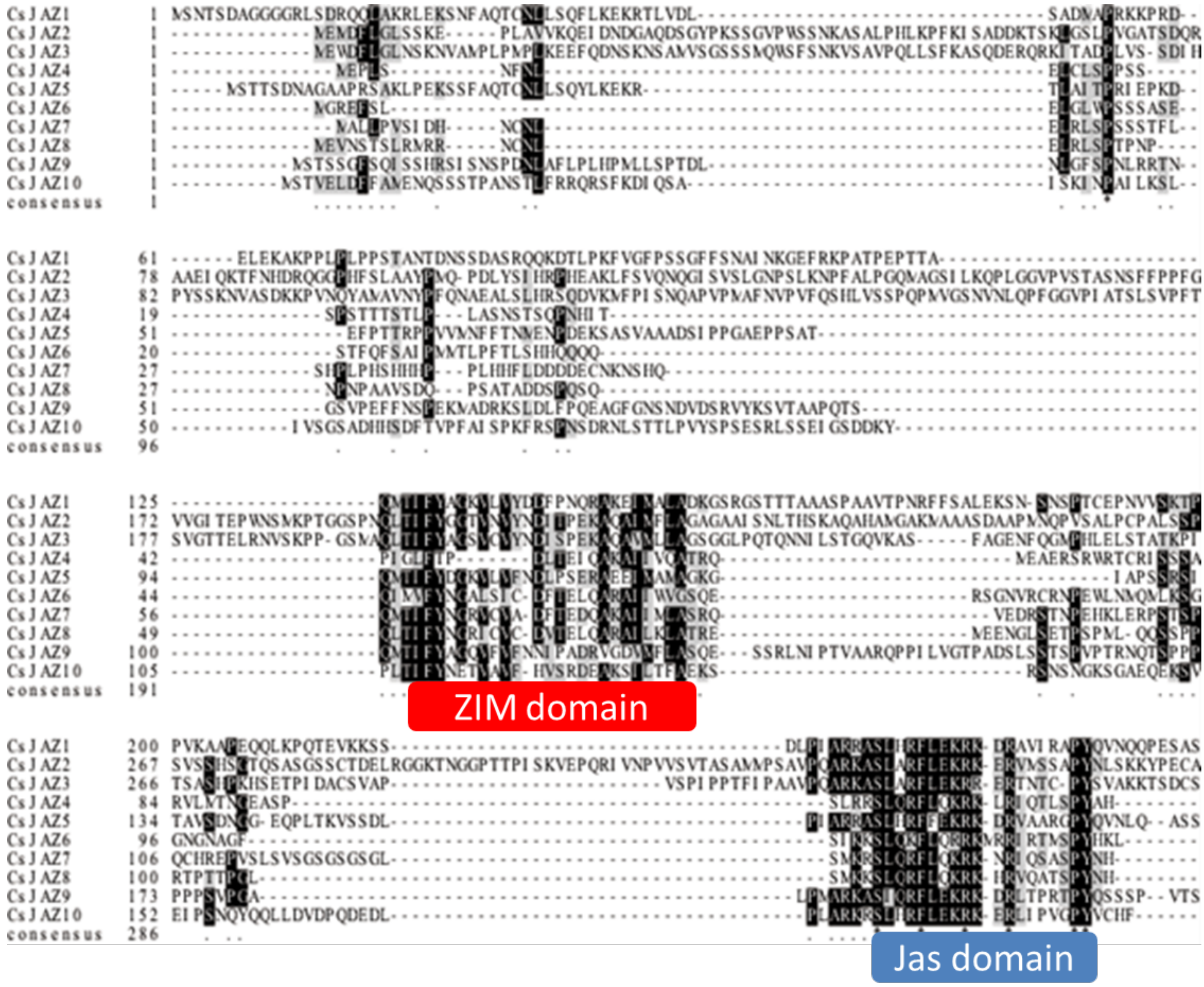

Supplemental Figure S10. Phylogenetic tree analysis of ten JAZs in cucumber.

The neighbor-joining phylogenetic tree with Poisson correction is based on amino acid sequence alignments. The phylogenetic tree was constructed using MEGA 5. Bootstrap analysis was employed using 5000 replicates. CsJAZs are marked with ●. The JAZs GenBank accession numbers are listed in Supplemental Figure Table S4.

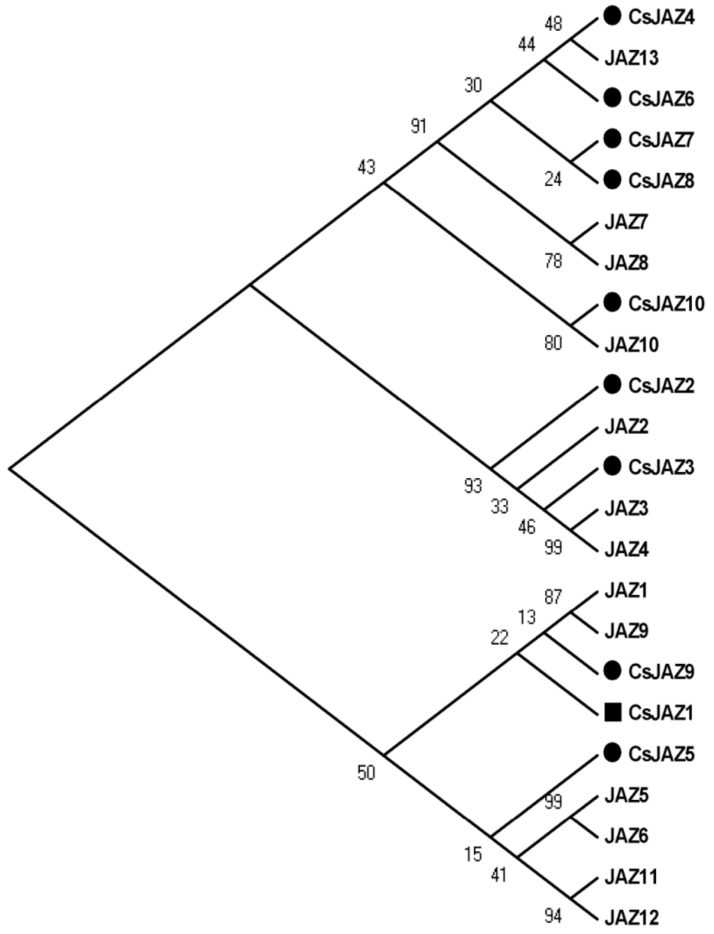

Supplemental Table S1: Primers used in this study

Primer-F (for qRT-PCR)	Primer-F sequence in cucumber	Primer-R (for qRT-PCR)	Primer-R sequence in cucumber
qCsGL2-LIKE-F	TCGTTGGATTTCCTTCGTCG	qCsGL2-LIKE-R	CCGTTTGTGGTTTCAGTTGCT
qCsJAZ1-F	TCGTTGGATTTCCTTCGTCG	qCsJAZ1-R	CCGTTTGTGGTTTCAGTTGCT
qCsJAZ2-F	GGTTCCGTTGTCGGGATTACT	qCsJAZ2-R	GGATGCTTTGCGAGCCTGT
qCsJAZ3-F	AGGAAAATCACTGCTGACCCAC	qCsJAZ3-R	CACTGCCAGCTAAAAGCATAACA
qCsJAZ4-F	TTACCGAGATCCAGGCAAAGG	qCsJAZ4-R	TTAGTGAGCGTAGGGTGAAAGTGT
qCsJAZ5-F	CAAAAGATGAGTTTCCGACGACT	qCsJAZ5-R	CACTTGGTATGGACCTCTTGCTG
qCsJAZ6-F	TTCAGTTTGGAGCTTGGTCTTTG	qCsJAZ6-R	TCTGGGTTCCTACATCTCACATTTC
qCsJAZ7-F	TTGGAACTTCGTCTTTCTCCTTCT	qCsJAZ7-R	TGGATCTGTCCTCGACCTGTCT
qCsJAZ8-F	ATGGAAGTAAATTCCACCTCTCTCA	qCsJAZ8-R	TCAATGGTTGTATGGAGAAGTTGCT
qCsJAZ9-F	GGCGGATCGGAAGTCTTTG	qCsJAZ9-R	GGCGTTCTCGGAGTTAGTCTGT
qCsJAZ10-F	CATCACTCCGATTTCACTGTTCC	qCsJAZ10-R	CAACGTCCAGGAGTTGTTGGTAC
qCsFT-F	AATCAACCAAGAGTCGAGATTGG	qCsFT-R	TTGCACCTGTTGTAGCTGGAATA
qCsLFY-F	ACTCGGATTCACCGTCAGCA	qCsLFY-R	CTAGACGTCTGCGTTCGGCT
qCsAG-F	ATGTGAACATGATGGGAGGAGAA	qCsAG-R	TTGAAGAGCCATGTTGTCTTGG
qCsCO-F	CCAGACCCAACGCCAGTTATT	qCsCO-R	CCCCAGTTTGTCCTCCCATT
qCsAP1-F	GGAGAAGATTTGGATTCGTTGAGT	qCsAP1-R	CATTGTAACCCTGCTGTTGCTG
qCsAP3-F	CTGCCACTTCGACTAAGGAGCT	qCsAP3-R	GGTACTTGCGTTCACGGATGAT
qCsELF3-F	TAAAGAAAGCTGGACTTCGGTCA	qCsELF3-R	CGACGACAACATCAGGGGAG
qCsAOC2-F	CCACCTCTCCTCTGCTCTCT	qCsAOC2-R	CTGTAGATGGCCTCGTAGCG
qCsLOX2-F	GGCAGTTGGCTACTGTCCTT	qCsLOX2-R	ATGCTCCCTCATCCAGAGTC
qCsLOX3-F	GTTCTCGATCAAAGCGCGTC	qCsLOX3-R	ATGAGGCTTTGAAGGGTCCG
Actin-F	TCGTGCTGGATTCTGGTG	Actin-R	GGCAGTGGTGGTGAACAT
**Primer-F**	**Primer-F sequence**	**Primer-R**	**Primer-R sequence**
ACTIN2 (Ara) -F	GATGATGCGCCAAGAGCTG	ACTIN2 (Ara) -R	GCCTCATCACCTACGTAGGCAT
AtGL2(SmaI)-F for transformation	cccgggATGAAGTCGA TCGATGGCTG	AtGL2(SpeI)-R for transformation	actagtTCAGCAATCTTCGATTTGTAGAC
**Primer-F**	**Primer-F sequence**	**Primer-R**	**Primer-R sequence**
CsGL2-LIKE-F for clone	ATGGGTGCCGACATGTCCAA	CsGL2-LIKE-R for clone	TTAATCCTCCTCACAACACATGCTG
CsJAZ1-F	ATGTCGAATACATCGGACGC	CsJAZ1-R	TCATAAGTTGAGATCCAGATGCCT
**Primer-F for RNAi vector**	**Primer-F sequence**	**Primer-R for RNAi vector**	**Primer-R sequence**
CsGL2-LIKE(AscI)-F	ggcgcgccCGAGCAATTACGTATTAAGA ATGCC	CsGL2-LIKE(SwaI)-R	attaatTCCGGACCCAAAGTGGGTC
CsGL2-LIKE(BamHI)-F	ggatccTCCGGACCCAAAGTGGGTC	CsGL2-LIKE(SpeI)-R	actagtCGAGCAATTACGTATTAAGAATG CC
**Primer-F for BiFC**	**Primer-F sequence**	**Primer-R for BiFC**	**Primer-R sequence**
CsGL2-LIKE(AcsI)-F	ggcgcgccATGGGTGCCGACATGTCCAA	CsGL2-LIKE(BamHI)-R	ggatccATCCTCCTCACAACACATGCTG
CsMYC2(AcsI)-F	ggcgcgccATGTTACTTATGGATGAAATCAAT ATTTC	CsMYC2(BamHI)-R	ggatccTTCAAGTCTTTGAAGTATGGCTGTT
CsJAZ1(AscI)-F	ggcgcgccATGTCGAATACATCGGACGC	CsJAZ1(BamHI)-R	ggatccTAAGTTGAGATCCAGATGCCT
**Primer-F for Co-IP /GFP**	**Primer-F sequence**	**Primer-R for Co-IP/GFP**	**Primer-R sequence**

CsGL2-LIKE(SmaI)-F	cccgggATGGGTGCCGACATGTCCAA	CsGL2-LIKE(SpeI)-R	actagtATCCTCCTCACAACACATGCTG
CsJAZ1(EcoRI)-F	actagtATGTCGAATACATCGGACGC	CsJAZ1(BamHI)-R	gaattcAGCGTAGTCTGGGACGTCGTATGGG
CsJAZ1(SmaI)-F	cccgggATGTCGAATACATCGGACGC	CsJAZ1(SpeI)-R	actagtTAAGTTGAGATCCAGATGCCT
**Primer-F for ectopic expression**	**Primer-F sequence**	**Primer-R for ectopic expression**	**Primer-R sequence**
CsJAZ1-1-F	ATGTCGAATACATCGGACGC	CsJAZ1-1-R	AGCCCTCCTAGCAATAGGCA
CsJAZ1-2-F	CAAGTAAACCAACAGCCAGAGTC	CsJAZ1-2-R	TAAGTTGAGATCCAGATGCCTTG
CsJAZ1△Jas(SmaI)-F	cccgggATGTCGAATACATCGGACGC	CsJAZ1△Jas(SpeI)-R	actagtTAAGTTGAGATCCAGATGCCTTG
**Primer-F for H2Y**	**Primer-F sequence**	**Primer-R for H2Y**	**Primer-R sequence**
CsGL2-LIKE(NdeI)-F	catatgATGGGTGCCGACATGTCCAA	CsGL2-LIKE(EcoRI)-R	gaattcATCCTCCTCACAACACATGCTG
CsGL2-LIKE-HD(NdeI)-F	catatgATGGGTGCCGACATGTCCAA	CsGL2-HD(EcoRI)-R	gaattcATTTTCAAGTCCAAATATTCCCGTG
CsGL2-START(NdeI)-F	catatgTCAAGAATCATGGAGAAAGTTGATG	CsGL2-START(EcoRI)-R	gaattcATCCTCCTCACAACACATGCTG
CsHDG1(NDEI)AD-F	catatgATGGACTTTGGCGGTGGCT	CsHDG1(ECORI)AD-R	gaattcGGAACTATGACAATTTAAGGCTGTCTTG
CsHDG2(NDEI)AD-F	catatgATGTTTGGAGCACATGGATTTGAAG	CsHDG2(ECORI)AD-R	gaattcATTGATGGGTTGGTCACACATGAGT
CsHDG3(NDEI)AD-F	catatgATGGCAAGTAATAACCTAGAAAGCAAT	CsHDG3(ECORI)AD-R	gaattcAAATTGTTGAATATTGAAAGCCCCT
CsHDG4(NDEI)AD-F	catatgATGTTCCAGCCAAGCATTATGG	CsHDG4(ECORI)AD-R	gaattcTGCATTCTCACATGATAATGAAGC
CsHDG5(NDEI)AD-F	catatgATGCCTGGTGGTGCAATTACTGATC	CsHDG5(ECORI)AD-R	gaattcCGATACTTGAAGGGCAGCTTTGATC
CsHDG6(NDEI)AD-F	catatgATGGATATTATGATGGATGGTCGTG	CsHDG6(ECORI)AD-R	gaattcATCCAAACAAGAGCAATTCAATGC
CsGL3(NDEI)AD-F	catatgATGTTCGATCCAGATATGTTTGATA	CsGL3(ECORI)AD-R	gaattcTGGGTTTTCACGCATCACAG
CsHDG7(NDEI)AD-F	catatgATGAGTTTTGGGGGTTTCCTCGAC	CsHDG7(ECORI)AD-R	gaattc TCAGGTTTCGCATTGAAGAGCG
CsHDG8(NDEI)AD-F	catatgATGCCAGCCGGAATGATGATT	CsHDG8(ECORI)AD-R	gaattcAGGATTGTCGCATGACAAAGAGG
CsHDG9(NDEI)AD-F	catatgATGGCCGTTGTCATGTCCGA	CsHDG9(ECORI)AD-R	gaattcATCATCTTCACCTTGTAGGCTTGCTT
CsJAZ2(NDEI)AD-F	catatgATGGAGATGGATTTCTTGGGTT	CsJAZ2(ECORI)AD-R	gaattcGCTTGCCACATTAGCGCTG
CsJAZ3(NDEI)AD-F	catatgATGGAGTGGGATTTTTTGGGT	CsJAZ3(ECORI)AD-R	gaattcGGCCATCAGATCCAAGGTTG
CsJAZ4(NDEI)AD-F	catatgATGGAGATGGATTTCTTGGGTTTG	CsJAZ4(ECORI)AD-R	gaattcGCTTGCCACATTAGCGCTG
CsJAZ5(NDEI)AD-F	catatgATGTCTACTACCTCCGACAACGC	CsJAZ5(ECORI)AD-R	gaattcATTTAAATCAAACTGCTTCGAAGATT
CsJAZ6(NDEI)AD-F	catatgATGGGAAGGGAGTTCAGTTTG	CsJAZ6(ECORI)AD-R	gaattcCAGTTTGTGATATGGAGACATTGTT
CsJAZ7(NDEI)AD-F	catatgATGGCCCTTCTCCCCGTTT	CsJAZ7(ECORI)AD-R	gaattcATGATTGTAAGGTGAAGCAGATTGAA
CsJAZ8(NDEI)AD-F	catatgATGGAAGTAAATTCCACCTCTCTCA	CsJAZ8(ECORI)AD-R	gaattcATGGTTGTATGGAGAAGTTGCT
CsJAZ9(NDEI)AD-F	catatgATGTCGACTTCTTCCGGATTCTCTC	CsJAZ9(ECORI)AD-R	gaattcTTTTTGAGACTGCACCGCCAAT
CsJAZ10(NDEI)AD-F	catatgATGTCCACTGTTGAACTCGATTTCTT	CsJAZ10(ECORI)AD-R	gaattcGAAATGACAAACATAAGGCCCCAC
CsJAZ1(NDEI)-F	catatgATGTCGAATACATCGGACGC	CsJAZ1(EcoRI)-R	gaattcTAAGTTGAGATCCAGATGCCT
CsJAZ1(NDEI)△Jas-F	catatgATGTCGAATACATCGGACGC	CsJAZ1(EcoRI)△Jas-R	gaattcAGCCCTCCTAGCAATAGGCAA
CsJAZ1(NDEI)△ZIM-F	catatgGATAAAGGAAGCCGCGGC	CsJAZ1(EcoRI)△ZIM-R	gaattcTAAGTTGAGATCCAGATGCCT
**Primer-F for in suit**	**Primer-F sequence**	**Primer-R for in suit**	**Primer-R sequence**
CsGL2-LIKE-SP6-F	GATTTAGGTGACACTATAGaatGCTGTTCTGCTTTCTTCATGGCAAC	CsGL2-LIKE-T7-R	tgTAATACGACTCACTATAGGGTTAATCCTCCTCACAACACATGCT
CsJAZ1-SP6-F	GATTTAGGTGACACTATAGaatGCTATGTCGAATACATCGGACGCC	CsJAZ1-T7-R	tgTAATACGACTCACTATAGGGTCATAAGTTGAGATCCAGATGCCTT

Supplemental Table S2：Genes involved in pollen and pollen tube development in DEGs of CsGL2-LIKE RNAi plants

Gene	Gene Description	log2FC	P-value
Csa3G006660	PHD finger protein	-18.22116	6.67E-183
Csa5G291670	PHD finger protein MALE STERILITY 1	-17.76914	6.34E-196
Csa5G291680	PHD finger protein	-12.63363	6.11E-121
Csa2G108690	Cytochrome P450, putative	-10.334	1.15E-195
Csa5G589320	4-coumarate:CoA ligase-like	-9.430865	1.33E-194
Csa4G551130	Putative fatty acyl coA reductase	-8.332835	5.25E-181
Csa3G119520	Cytochrome P450	-8.135877	8.33E-177
Csa6G116160	Dihydroflavonol 4-reductase family	-8.081777	1.54E-180
Csa3G172380	Glucan endo-1,3-beta-glucosidase, putative	-7.972839	1.20E-176
Csa5G609630	Cinnamoyl-CoA reductase	-7.239974	7.99E-189
Csa4G358690	Chalcone synthase	-5.656246	3.09E-175
Csa4G358690	Chalcone synthase	-5.654912	2.16E-188
Csa5G198190	Glucan endo-1,3-beta-glucosidase	-3.992981	1.07E-175
Csa6G526410	Polygalacturonase QRT3	-3.135888	1.27E-187
Csa1G006310	Chalcone synthase, putative	-2.680411	2.44E-183
Csa6G423450	ER glycerol-phosphate acyltransferase	-2.344853	3.27E-158
Csa5G605750	Mads box protein, putative	2.4096749	1.11E-120
Csa4G496250	MADS-box transcription factor family protein	17.645149	1.90E-120

Supplemental Table S3：List of proteins that interacted with CsGL2-LIKE

Accession Number	Description
Csa1G165210.1	F1F0-ATPase inhibitor protein
Csa1G445860.1	Chlorophyll a-b binding protein 3
Csa2G006240.1	Bundle-sheath defective protein 2 family
Csa3G164500.1	Ring finger protein
Csa3G271350.1	Putative S-adenosylmethionine decarboxylase
Csa5G576650.3	Phosphatidylserine synthase
Csa6G139750.1	Chlorophyll a-b binding protein
Csa7G398150.1	Ribosomal protein L19
Csa7G448810.1	Jasmonate ZIM domain protein h

Supplemental Table S4: Genes information used in this study

Genes name	Species	Accession number
CsGL2-LIKE	*Cucumis sativus*	KM095653.1
CsJAZ1	*Cucumis sativus*	Csa7G448810
CsJAZ2	*Cucumis sativus*	Csa5G628650
CsJAZ3	*Cucumis sativus*	Csa1G042920
CsJAZ4	*Cucumis sativus*	Csa1G435790
CsJAZ5	*Cucumis sativus*	Csa3G645940
CsJAZ6	*Cucumis sativus*	Csa4G062400
CsJAZ7	*Cucumis sativus*	Csa1G435720
CsJAZ8	*Cucumis sativus*	Csa6G523460
CsJAZ9	*Cucumis sativus*	Csa1G597690
CsJAZ10	*Cucumis sativus*	Csa4G009880
CsFT	*Cucumis sativus*	Csa1G651710
CsLFY	*Cucumis sativus*	Csa1G000050
CsTFL	*Cucumis sativus*	Csa6G452100
CsAG	*Cucumis sativus*	Csa6G520410
CsAP1	*Cucumis sativus*	Csa6G367090
CsAP3	*Cucumis sativus*	Csa3G865440
CsELF3	*Cucumis sativus*	Csa7G452030
CsCO	*Cucumis sativus*	Csa1G420310
CsLOX2	*Cucumis sativus*	Csa4G288610
CSLOX3	*Cucumis sativus*	Csa7G449420
CsAOC2	*Cucumis sativus*	Csa7G449420
AtANL2	*Arabidopsis thaliana*	AT4G00730
AtATML1	*Arabidopsis thaliana*	AT4G21750
AtGL2	*Arabidopsis thaliana*	AT1G79840
AtHDG1	*Arabidopsis thaliana*	AT3G61150
AtHDG2	*Arabidopsis thaliana*	AT1G05230
AtHDG3	*Arabidopsis thaliana*	AT2G32370
AtHDG4	*Arabidopsis thaliana*	AT4G17710
AtHDG5	*Arabidopsis thaliana*	AT5G46880
AtHDG6	*Arabidopsis thaliana*	AT4G25530
AtHDG7	*Arabidopsis thaliana*	AT5G52170
AtHDG8	*Arabidopsis thaliana*	AT3G03260
AtHDG9	*Arabidopsis thaliana*	AT5G17320
AtHDG10	*Arabidopsis thaliana*	AT1G34650
AtHDG11	*Arabidopsis thaliana*	AT1G73360
AtHDG12	*Arabidopsis thaliana*	AT1G17920
AtPDF2	*Arabidopsis thaliana*	AT4G04890
JAZ1	*Arabidopsis thaliana*	AT1G19180
JAZ2	*Arabidopsis thaliana*	AT1G74950
JAZ3	*Arabidopsis thaliana*	AT3G17860
JAZ4	*Arabidopsis thaliana*	AT1G48500
JAZ5	*Arabidopsis thaliana*	AT1G17380
JAZ6	*Arabidopsis thaliana*	AT1G72450
JAZ7	*Arabidopsis thaliana*	AT2G34600
JAZ8	*Arabidopsis thaliana*	AT1G30135

Genes name	*Species*	Accession number
JAZ9	*Arabidopsis thaliana*	AT1G70700
JAZ10	*Arabidopsis thaliana*	AT5G13220
JAZ11	*Arabidopsis thaliana*	AT3G43440
JAZ12	*Arabidopsis thaliana*	AT5G20900
JAZ13	*Arabidopsis thaliana*	AT3G22275
AaHD1	*Artemisia annua*	KU744599.1
ZmOCL4	*Zea mays*	NM_001350876.1
GhHOX1	*Gossypium hirsutum*	NM_001327139.1
GhHOX3	*Gossypium hirsutum*	XM_016828890.1
OsROC5	*Oryza sativa*	AB101648.1
Slwo	*Solanum lycopersicum*	XM_010318312.3
